# The Epstein-Barr virus EBNA1 protein modulates the alternative splicing of cellular genes

**DOI:** 10.1186/s12985-019-1137-5

**Published:** 2019-03-04

**Authors:** Simon Boudreault, Victoria E. S. Armero, Michelle S. Scott, Jean-Pierre Perreault, Martin Bisaillon

**Affiliations:** Département de biochimie, Faculté de médecine et des sciences de la santé, Université de Sherbrooke, Sherbrooke, Québec J1E 4K8 Canada

**Keywords:** Alternative splicing, Epstein-Barr virus, Virus-host interaction, High-throughput RT-PCR, RIP-sequencing, EBNA1, Splicing factors

## Abstract

**Background:**

Alternative splicing (AS) is an important mRNA maturation step that allows increased variability and diversity of proteins in eukaryotes. AS is dysregulated in numerous diseases, and its implication in the carcinogenic process is well known. However, progress in understanding how oncogenic viruses modulate splicing, and how this modulation is involved in viral oncogenicity has been limited. Epstein-Barr virus (EBV) is involved in various cancers, and its EBNA1 oncoprotein is the only viral protein expressed in all EBV malignancies.

**Methods:**

In the present study, the ability of EBNA1 to modulate the AS of cellular genes was assessed using a high-throughput RT-PCR approach to examine AS in 1238 cancer-associated genes. RNA immunoprecipitation coupled to RNA sequencing (RIP-Seq) assays were also performed to identify cellular mRNAs bound by EBNA1.

**Results:**

Upon EBNA1 expression, we detected modifications to the AS profiles of 89 genes involved in cancer. Moreover, we show that EBNA1 modulates the expression levels of various splicing factors such as hnRNPA1, FOX-2, and SF1. Finally, RNA immunoprecipitation coupled to RIP-Seq assays demonstrate that EBNA1 immunoprecipitates specific cellular mRNAs, but not the ones that are spliced differently in EBNA1-expressing cells.

**Conclusion:**

The EBNA1 protein can modulate the AS profiles of numerous cellular genes. Interestingly, this modulation protein does not require the RNA binding activity of EBNA1. Overall, these findings underline the novel role of EBNA1 as a cellular splicing modulator.

**Electronic supplementary material:**

The online version of this article (10.1186/s12985-019-1137-5) contains supplementary material, which is available to authorized users.

## Background

Alternative splicing (AS) is an important mechanism allowing higher proteome diversity in eukaryotes. In *Homo sapiens*, AS is nearly ubiquitous, as more than 90% of human genes undergo AS [1]. AS, as opposed to constitutive splicing, leads to different arrangement of exons, retained introns, and splice-sites for the same pre-messenger RNA (pre-mRNA). This allows the same pre-mRNA to be processed into different isoform-coding mature mRNAs, sometimes even with opposing functions at the protein level. The regulatory aspect of AS is becoming increasingly known, and changes in AS are linked with various diseases such as cancer, Parkinson’s disease, amyotrophic lateral sclerosis, and rheumatoid arthritis [2–5].

Recently obtained evidence show that viruses can disrupt the AS of cellular transcripts, although functional consequences on viral infection are still sparse. A number of studies have shown different mechanisms allowing viruses from various families to alter host-cell AS. For example, Sindbis virus sequestration of HuR protein in the cytoplasm through multiple HuR 3′-UTR binding sequences in viral genomic and subgenomic RNAs modifies the splicing of *PCBP2* and *DST* transcripts (a complete list of all gene names used in this manuscript and their official complete name is included in Additional file 1: Table S1) [6]. Similarly, the poliovirus protease 2A (2Apro) is able to change the cellular localization of HuR, TIA1 and TIAR, thereby impacting the splicing of the apoptotic gene *FAS* [7]. Moreover, Vesicular stomatitis virus (VSV) infection triggers relocalization of splicing factors, such as hnRNPA1, hnRNPC1/C2, and hnRNPKM from the nucleus to the cytoplasm with functional consequences on splicing still unknown [8]. Finally, several transcriptomics studies have shown dysregulation of AS in different cell models following viral infection [9–11]. How the course of viral infection is affected by these AS modifications remains to be investigated.

Studies about the impact of *herpesviridae* on cellular splicing have mainly focused on Herpes simplex virus 1 (HSV-1) and its immediate early ICP27 protein. ICP27 interferes with the splicing machinery and thus contributes to host protein synthesis shut-off during HSV-1 infection [12, 13]. The interaction of ICP27 with components of the splicing machinery and the redistribution of splicing factors are involved in this phenomenon [14, 15]. However, RNA-sequencing studies performed on HSV-1 infected cells found no evidence of disruption of AS upon viral infection [16]. New evidence on the modulation of AS by Epstein-Barr virus (EBV), another virus from the *herpesviridae* family involved in numerous cancers (such as Burkitt’s and Hodgkin’s lymphomas, gastric carcinoma, and nasopharyngeal cancer), is emerging and suggests potential roles for the EBV SM protein and EBER1/2 non-coding RNAs in this modulation of AS. The viral SM protein was shown to alter the splicing of *STAT1* gene, resulting in a transcript coding for a dominant-negative STAT1 protein [17, 18]. Because of the role of STAT1 in the interferon signal transduction pathways, the impact of the altered splicing on infectivity is presumably important. Moreover, the two non-coding RNAs (EBER1 and EBER2), which accumulate in the nucleus during latent infection can alter cellular AS. EBER1 was shown to interact with the AUF1/hnRNP D splicing factor, and expression of both EBERs in cultured cell line leads to significant changes in the AS landscape of the host-cell [19, 20].

Aside from EBERs and various mi-RNAs, EBNA1 is the only protein expressed in all forms of latency during EBV infection [21]. It localizes to the nucleus and has multiple roles including binding to cellular and viral genomes, regulation of signaling pathways, and gene transcription [22, 23]. EBNA1 is also thought to act as an oncoprotein and links EBV infection to carcinogenesis. For example, EBNA1 can bind USP7, a ubiquitin protease stabilizing both p53 and MDM2. This competitive binding destabilizes both p53 and MDM2, thus promoting cell survival [24]. Furthermore, EBNA1 increases the levels of STAT1, the turnover of SMAD2, and inhibits the activity and DNA binding properties of NF-κB; all of these proteins have well-known roles in tumorigenesis [25, 26]. The oncogenic properties of the EBNA1 have been discussed elsewhere [27].

Recently, we demonstrated that EBNA1 expression modulates the AS of various cellular genes which are also dysregulated in EBV-positive gastric carcinomas [28]. In the present study, we present the mechanistic investigation behind those changes and possible causes underlying this modulation. The ability of EBNA1 to modify the AS of 1238 cellular genes involved in cancer was investigated using a high-throughput RT-PCR platform. Our results highlight viral modulation of AS as a potential key player in tumorigenesis.

## Methods

### Generation of stable EBNA1 HEK293T cells

Stable HEK293T cells expressing the EBNA1-FLAG-HA protein were generated using MSCV-N EBNA1 plasmid. MSCV-N EBNA1 was a gift from Karl Munger (Addgene plasmid # 37954) [29]. Upon transfection, cells were selected using 5 μg/mL puromycin for 20 days. To maintain the selection, cells were kept in 3 μg/ml puromycin-DMEM-10% fetal bovine serum.

### Validation of EBNA1 expression by Western blot

HEK293T cells and HEK293T stable cells expressing EBNA1-FLAG-HA were grown upon confluency in T-75 flasks, trypsinized and pelleted at 1500 rpm, 5 min. Cell were resuspended in RIPA Buffer (1% Triton X-100, 1% sodium deoxycholate, 0.1% SDS, 1 mM EDTA, 50 mM Tris-HCl pH 7.5 and complete protease inhibitor (ROCHE)) and lysed using ultrasound on ice at 13% amplitude, 5 s for two times. Cellular debris were then pelleted at 13000 RPM, 4 °C, 10 min. If chromosomic DNA was still floating after centrifugation, the ultrasound and centrifugation process was carried out a second time. Lysates were dosed for total protein in triplicate using standard Bradford assay (Thermo Scientific Coomassie Protein Assay). The appropriate quantity of protein was diluted to ten microliters in water, completed with Laemmli buffer at 1x (final concentration) and heated 5 min at 95 °C. Sample were loaded on 10% SDS-polyacrylamide gels and electrophoresis on was carried out at 150 V. Gels were transferred onto a polyvinylidene difluoride (PVDF) membrane at 4 °C, 1 h15, 100 V. The membrane was blocked in 5% milk in TBS-T (10 mM Tris-HCl pH 8.0, 220 mM NaCl, 0.1% Tween 20), 1 h at room temperature. Upon washing in TBS-T (3x, 5 min each), mouse anti-Flag antibody (Sigma Aldrich), anti-HA or anti-EBNA1 antibody (Santa Cruz Biotechnology) diluted 1:1000 (Flag, HA) or 1:100 (EBNA1) in 2.5% milk/PBS were incubated overnight with the membrane in humid chamber at 4 °C. The membrane was washed 3 times in TBS-T and incubated with a sheep anti mouse-HRP secondary antibody 1:5000 (Amersham Biosciences) during 1 h in a humid chamber at room temperature. Membrane was washed 3 times with TBS-T and one time with PBS. Bound antibodies were revealed using an enhanced chemiluminescence (ECL) kit (Perkin Elmer) and scanned on ImageQuant LAS4000 (GE Healthcare Life Science). Mouse anti-β-actin loading control (Sigma) was done on the same membrane after stripping the membrane by boiling it in PBS for 1 min. The procedure was then the same from the blocking upon revelation using the anti-β-actin antibody diluted 1:2000 (in 2.5% milk/PBS) and the secondary anti-mouse (1:5000) antibody from Amersham.

### Immunofluorescence of stably expressing EBNA1 HEK293T

The day before, HEK293T cells stably expressing the EBNA1-FLAG-HA protein were seeded at a density of 5 × 104 cells/well in 24-well plates. Cells were washed twice with PBS and fixed 20 min using 4% paraformaldehyde and 4% sucrose in PBS at room temperature. Cells were then permeabilized with 0.15% triton X-100 in PBS for 5 min at room temperature and blocked in 10% normal goat serum (Wisent). Anti-HA antibody (Santa Cruz Biotechnologies) was incubated 4 h at room temperature to allow detection of EBNA1-FLAG-HA protein. Cells were washed and incubated 1 h in the dark with DyLight 488-labelled goat anti-mouse secondary antibody (ThermoFisher Scientific). Nucleus staining was performed using 1 μg/ml Hoechst, 15 min at room temperature. Cover glasses were mounted on slides with SlowFadeGold mounting medium (Life Technologies), then epifluorescence microscopy was conducted using a Nikon Eclipse TE2000-E visible/epifluorescence inverted microscope using bandpass filters for Hoechst and DyLight 488.

### High throughput RT-PCR screening of AS events

HEK293T and HEK293T cells expressing EBNA1-FLAG-HA were grown upon confluency and pelleted. Total RNA extractions were performed on cell pellets using TRIzol (Invitrogen) with chloroform, following the manufacturer’s protocol. The aqueous layer was recovered, mixed with one volume of 70% ethanol and applied directly to a RNeasy Mini Kit column (Qiagen). DNAse treatment on the column and total RNA recovery were performed as per the manufacturer’s protocol. RNA integrity was assessed with an Agilent 2100 Bioanalyzer (Agilent Technologies). Reverse transcription was performed on 2.2 μg total RNA with Transcriptor reverse transcriptase, random hexamers, dNTPs (Roche Diagnostics), and 10 units of RNAse OUT (Invitrogen) following the manufacturer’s protocol in a total volume of 20 μl. All forward and reverse primers were individually resuspended to 20–100 μM stock solution in Tris-EDTA buffer (IDT) and diluted as a primer pair to 1.2 μM in RNase DNase-free water (IDT). End-point PCR reactions were done on 10 ng cDNA in 10 μL final volume containing 0.2 mmol/L each dNTP, 1.5 mmol/L MgCl2, 0.6 μmol/L each primer, and 0.2 units of Platinum Taq DNA polymerase (Invitrogen). An initial incubation of 2 min at 95 °C was followed by 35 cycles at 94 °C 30 s, 55 °C 30 s, and 72 °C 60 s. The amplification was completed by a 2 min incubation at 72 °C. PCR reactions were carried on thermocyclers GeneAmp PCR System 9700 (ABI), and the amplified products were analyzed by automated chip-based microcapillary electrophoresis on Caliper LC-90 instruments (Caliper LifeSciences). Amplicon sizing and relative quantitation was performed by the manufacturer’s software, before being uploaded to the LIMS database.

### String network

Using the STRING database [30] version 10.0, genes were submitted for generation of protein-protein interaction network from the *Homo sapiens* interactome. *P*-value for the protein-protein interaction enrichment was directly recovered from the STRING analysis.

### Gene ontology analysis

The Database for Annotation, Visualization and Integrated Discovery (DAVID) [31] version 6.8 with Bonferroni correction was used. Reference background was composed of all genes analysed in the high-throughput RT-PCR assay in order to take into account the bias from cancer-related genes.

### Protein levels of splicing factors

As described before, HEK293T cells and HEK293T stable cells expressing EBNA1-FLAG-HA were grown upon confluency, trypsinized and lysed in RIPA buffer. Lysates were dosed and loaded on 10% acrylamide gel. Antibodies were diluted 1:1000 for SF1 (Abgent), Rbm23 (Abgent); hnRNPA1 (Rabbit polyclonal against the ASASSSQRGR peptide, see [32]), Fox-2 (Abcam), and 1:100 for SRSF3 (Santa Cruz Biotechnologies). Secondary anti rabbit antibody (Cell Signaling Technology) diluted 1:5000 was used for the first 4 antibodies and an anti mouse (Cell Signaling Technology) diluted 1:5000 for the SRSF3 antibody. Results shown in this paper are representative results of two to three independent experiments.

### RNA immunoprecipation

HEK293T cells and HEK293T stable cells expressing EBNA1-FLAG-HA were grown upon confluency in P150 dishes, harvested and pelleted. They were then washed twice in cold PBS, and resuspended in the same volume as the pellet of polysome lysis buffer (10 mM Hepes pH 7.0, 100 mM KCl, 5 mM MgCl2, 0.5% NP-40, 1 mM DTT, 100 u/mL RNase inhibitor and complete protease inhibitor (Roche)). After an incubation of 5 min on ice, they were frozen at − 80 °C to complete the lysis. After a rapid thaw, tubes were centrifuged 20 min at 4 °C, 13000 g. The supernatant was dosed using the Bradford assay (Thermo Scientific Coomassie Protein Assay) in triplicate. For each IP, 50 μL of Anti-Ha matrix (Roche) was centrifuged 1 min at 13,400 rpm and the supernatant was discarded. Beads were washed with 1 mL of NT2 buffer (50 mM Tris-HCl, pH 7.4, 150 mM NaCl, 1 mM MgCl2, 0.05% NP-40) followed by 1 min centrifugation at 13,400 rpm for 5 times. They were then resuspended in 900 μL of NET-2 buffer (NT2 buffer supplemented with 20 mM EDTA, pH 8.0, 1 mM DTT and 100 U/mL RNase inhibitor) and 100 μL (adjusted with NT-2 buffer to a total protein quantity of 2.5 mg) of lysate from either control or EBNA1 expressing cells was added. Tubes were inverted a couple of times, centrifuged 1 min at 13,400 rpm, 4 °C and 100 μL was removed as the input to evaluate RNA degradation. All tubes were incubated on a rotating wheel at 4 °C overnight. Beads were precipitated at 5000 g for 5 min, 4 °C and washed five times with ice-cold NT2 buffer. Upon the last washing, beads were resuspended in 90 μL of NT-2 buffer and 10 μL of RQ1 DNase and incubated at 37 °C for 30 min. Input tubes were directly added 10 μL of RQ1 DNase. Dnase-treated IP were diluted with 1 mL NT-2 buffer, centrifuged and supernatant was discarded. Beads were resuspended in 150 μL of proteinase K buffer (1% SDS, 1.2 mg/mL proteinase K from Boehringer Mannheim), and input tubes were directly added SDS and proteinase K to the same volume. Tubes were incubated at 55 °C during 30 min with inversion at every 10 min. RNA was then extracted with phenol-chloroform, followed by a second chloroform extraction step. Precipitation of RNA using 272 mM ammonium acetate, 122 mM LiCl and 27 μg/mL glycogen in ice-cold ethanol was carried out during 2 h at − 80 °C and followed by high-speed centrifugation. Pellets were washed in 75% ethanol, ethanol was thoroughly removed, and RNA was resuspended in water.

### RIP-Seq library preparation

Quality (input) and quantity (input and IP) assessments were performed on Agilent Nano Chip (Catalog number 5067–1511). Inputs from both control and EBNA1 expressing cells both showed good RNA integrity (RIN = 8.9 and 9.2, respectively), thus underlining RNA was not degraded due to experimental procedures. RNA was ribodepleted using Illumina Ribo-Zero rRNA Removal Kit as per manufacturer’s protocol. The RNA-seq library was then built using Illumina SSV21106 kit from 9 μl ribo-depleted RNA. Library quality was assessed using Agilent DNA HS Chip (Catatalog number 5067–4626). Library quantification was performed by qPCR following Illumina Kappa library quantification protocol. HEK293T and HEK293T-EBNA1 libraries were multiplexed and sequencing was done using Illumina HiSeq 4000 at 100 bp paired-end reads at McGill University and Génome Québec Innovation Centre Sequencing Service.

### RIP-Seq analysis

Upon sequencing, 22,887,816 and 21,858,499 reads were obtained for control and EBNA1 RIP libraries, with respective average quality score of 39 and 38. Reads corresponding to both conditions were first trimmed and adaptors were removed using Trimmomatic (Galaxy Tool Version 0.32.2). STAR (version 2.5.1b) was used to align reads to hg38 human genome with annotation release 89 from the ENSEMBL database. Reads were sorted by names via samtools (version 1.3.2) and the rmdup function was used to remove PCR and sequencing duplicates. Reads unmapped or mapped to the scaffolds and their respective ID in the header were then remove using samtools (version 1.3.2) and the custom following awk command, because they are unsuitable for the analysis with RIPSeeker:

samtools view -h file.bam | awk ‘((NR < =197 && length($2) < 10) || (NR > =198 && length($3) < 5 && $3! ~ /[*]/)){print $0}’ > file.out.sam.

The output file was then converted to the bam format using samtools and analyzed using the R package RIPSeeker (version 1.10.0). The RIPSeeker package was chosen as it is one of the only programs specifically designed to assign peaks in RIP experiments [33]. RIPSeeker was used on both HEK293T-EBNA1 and HEK293T using HEK293T as a control to determine the reliability of assigned peaks.

### Data availability

The data discussed in this publication have been deposited in NCBI’s Gene Expression Omnibus [34] and are accessible through GEO Series accession number GSE107808 (https://www.ncbi.nlm.nih.gov/geo/query/acc.cgi?acc= GSE107808).

### qPCR

Reverse transcription was performed on 1.7 μg RNA (qPCR on splicing factors), 600 ng and 300 ng (RIP-qPCR, replicate 1 and 2–3, respectively) with Transcriptor reverse transcriptase, random hexamers, dNTPs (Roche Diagnostics), and 10 units of RNAse OUT (Invitrogen) following the manufacturer’s protocol in a total volume of 10 μl. All forward and reverse primers were individually resuspended to 20–100 μM stock solution in Tris-EDTA buffer (IDT) and diluted as a primer pair to 1 μM in RNase DNase-free water (IDT). Quantitative PCR (qPCR) reactions were performed in 10 μl in 96 well plates on a CFX-96 thermocycler (BioRad) with 5 μL of 2X iTaq Universal SYBR Green Supermix (BioRad), 10 ng (3 μl) cDNA (qPCR on splicing factors) or 5 ng (3 μl) cDNA (RIP-qPCR), and 200 nM final (2 μl) primer pair solutions. The following cycling conditions were used: 3 min at 95 °C; 50 cycles: 15 s at 95 °C, 30 s at 60 °C, 30 s at 72 °C. Relative expression levels were calculated using the qBASE framework. For every PCR run, control reactions performed in the absence of template were performed for each primer pair and these were consistently negative.

### As-PCR

Total RNA was extracted from cells using Qiazol (Qiagen) and following the manufacturer’s protocol. Reverse transcription was carried out using 1 μg of RNA and 4 μl of iScript Reverse Transcription Supermix (BIO-RAD) in a final volume of 20 μl using the following PCR program: 5 min at 25 °C; 20 min at 46 °C; 1 min at 95 °C. AS specific primer (IDT) were designed to amplify only one ASE and were resuspended together at 1 μM. Primers used and predicted amplicon are available in Additional file 1: Table S2. Each reaction was composed of ThermoPol buffer (NEB), dNTPs, primers, cDNA and TAQ (NEB) and were incubated using the following program: 2 min at 94 °C; 34 cycles: 30 s at 94 °C; 30 s at 55 °C; 1 min at 72 °C; 2 min at 72 °C (final elongation). PCR products were resolved using a Caliper LC-90 capillary electrophoresis (Caliper LifeSciences).

## Results

### Expression of EBNA1 in HEK293T

**Fig. 1:**
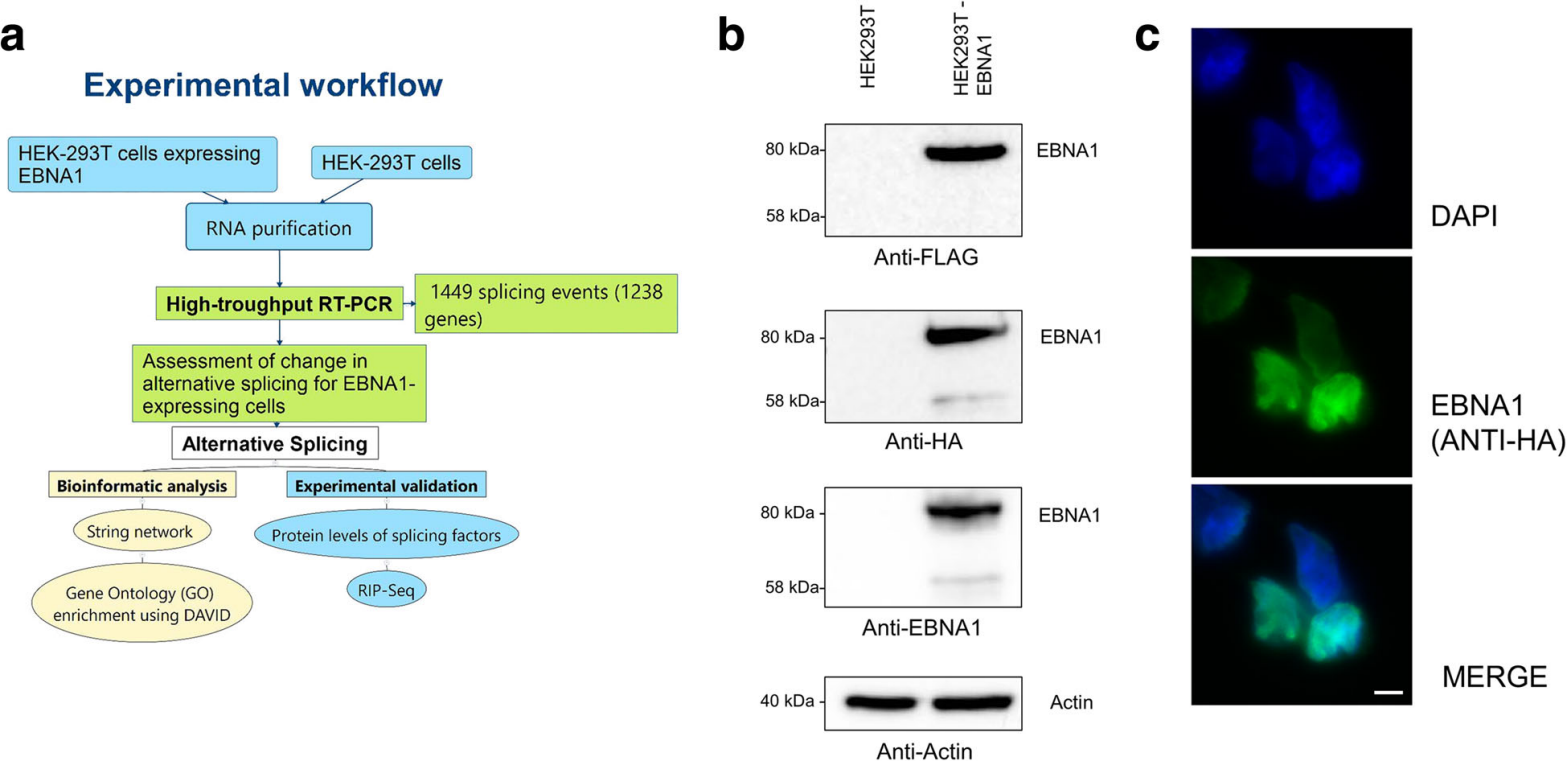
Experimental design and stable expression of the EBNA1 protein; (**a**) Overview of the strategy used to identify the changes in splicing in EBNA1-expressing cells. A high throughput RT-PCR approach was selected to look at the AS dynamics of numerous genes with a known involvement in cancer; (**b**) Western-Blot analysis showed expression of the EBNA1-HA-FLAG construct, as detected at 80- kDa by the anti-HA (1:1000), the anti-FLAG (1:1000) and the anti-EBNA1(1:100) antibodies upon stable selection of transfected HEK293T cells. Actin was used as a loading control on the same membrane after stripping. Each lane was charged with 20 μg of total protein. (**c**) Immunofluorescence of HEK293T-EBNA1-HA-FLAG cells using anti-HA antibody showed nuclear localization of the EBNA1-HA-FLAG protein, which confirm both the expression of the protein and its predicted cellular localization. Nuclei were stained using DAPI, and anti-HA and DAPI were images were merged. Scale bar, 10 μm

The EBNA1 protein is particularly interesting in regards to viral carcinogenesis as it is the only EBV protein expressed in all EBV-positive cancers [21]. Its constant expression in EBV-malignancies points toward a strong causative role. In the current study, the role of EBNA1 on cellular AS was investigated. Figure 1a outlines the global workflow used to study the impact of EBNA1 on AS. To decipher the role of EBNA1 on cellular AS, the HEK293T cell line was chosen since most of the mechanistic studies on EBNA1 have been performed in this cell line [23, 35–37]. A stable HEK293T cell line expressing the EBNA1 protein tagged with both HA/FLAG peptides was established using transfection. Upon antibiotic selection, EBNA1 expression was validated by Western blot using a antibodies against both the HA the FLAG tag, and using an EBNA1-specific antibody (Fig. 1b). This led to the detection of a major EBNA1 band at 80 kDa, consistent with previous reports [38–41]. EBNA1 expression was further validated in immunofluorescence, demonstrating a nuclear localization of the EBNA1-FLAG-HA construct, which is consistent with previous reports [22] (Fig. 1c).

### EBNA1 modulation of AS events

**Fig. 2:**
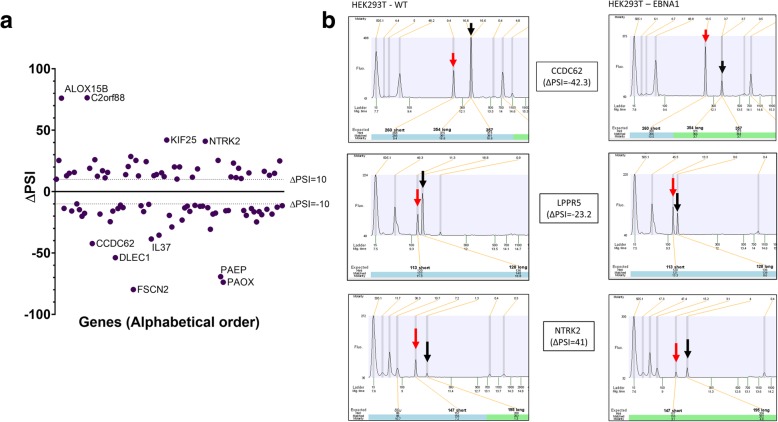
High-throughput RT-PCR shows expression of EBNA1 protein alters normal splicing of cellular genes implicated in cancer; (**a**) Respective ΔPSI values for the 89 genes that have their splicing modified in EBNA1-expressing cells. A cutoff of at least 10 of ΔPSI between control and EBNA1-expressing cells was used to identify changes in AS; (**b**) Relevant examples of splicing changes in EBNA1 expressing cells. The CCDC62, LPPR5 and NTRK2 transcripts all show important shift in the ASEs when EBNA1 is expressed. Red arrows indicate the short form for the ASE analyzed; black arrows indicate the long one

Evaluation of the ability of EBNA1 to modify the AS of cellular genes involved in cancer was performed by extracting total RNA from both control and EBNA1-expressing HEK293T cells. Following confirmation of the high quality of recovered RNA, it was subjected to a high-throughput RT-PCR platform designed to assess changes in AS. This assay has been developed to probe 1449 AS events affecting 1238 genes which are either suspected or have been confirmed to be involved in carcinogenesis [42]. Oligonucleotide primer pairs were designed to amplify specific AS events (ASEs), which generate amplicons of various lengths (either short or long depending on the status of the AS event). Following RT-PCR amplification, the products were resolved using capillary electrophoresis, detected, and quantified. The percent-spliced-in (PSI) metric was used to compare ASEs in both conditions (i.e. control cells and cells expressing EBNA1). ASEs with a high |ΔPSI| correspond to a significant modification of their splicing status when EBNA1 is expressed. Only ASEs with |ΔPSI| ≥ 10 were considered as being modified by EBNA1 expression. To ensure the right isoform choice from the electrophoregrams, all ASEs with high |ΔPSI| were manually curated. Upon visual inspection of electrophoregrams for accurate peak assignments, 89 ASEs had significant modifications to their splicing patterns attributable to the presence of EBNA1 (Fig. 2a; complete list in Table 1). For example, transcripts encoded by *CCDC62*, a nuclear receptor coactivator, and *LPPR5*, a membrane-bound signalization protein, both showed important shifts from the long form of the ASE to the short one in EBNA1-expressing cells (Fig. 2b). However, *NTRK2*, a tyrosine receptor kinase involved in the MAPK signaling pathway, shows a shift from the short to the long form in EBNA1-expressing cells. Additional representative electrophoregrams are depicted in supplementary data (Additional file 1: Figure S1). Finally, it should also be noted that using HEK293T cells transfected with an empty MSCV-N vector yielded similar results than using the control cell line (Additional file 1: Figure S2).

**Table 1:** List of genes that have their splicing modulated following EBNA1 expression with the respective ΔPSI for these AS events

Gene	ΔPSI	Gene	ΔPSI	Gene	ΔPSI
*ABTB1*	10.1	*FSCN2*	−79.8	*OPRL1*	−30.7
*ADAMTSL1*	25.4	*GCNT1*	25.4	*OSBPL9*	−18.3
*ALOX15B*	76.1	*GEM*	12.8	*OSCAR*	− 17.5
*ANKMY1*	−13.7	*GIGYF2*	−11.5	*P2RX5*	25.4
*AOC2*	12.9	*GK*	−16.3	*PAEP*	−69.3
*ASAH2B*	14.9	*HGF*	24.4	*PAOX*	−73.8
*ATG16L1*	−15.8	*ICA1L*	−10.4	*PAX2*	−15.6
*ATP11A*	15.7	*IL37*	−38.6	*PAXBP1*	−15.4
*ATP6V1C2*	−10.0	*IRF7*	13.0	*PCDH9*	12.0
*BMP4*	−14.7	*ITGA6*	13.9	*PHKB*	23.2
*BRD8*	−20.1	*ITGB1*	−35.5	*PITX2*	11.4
*C16orf13*	−17.7	*ITPR1*	15.3	*PKD1L2*	19.0
*C2orf88*	76.4	*IZUMO4*	12.2	*PLOD2*	10.7
*CAPN9*	19.0	*KIF25*	42.0	*PRC1*	−15.4
*CCDC62*	−42.3	*KIF9*	−19.4	*PTP4A3*	−19.4
*CD37*	26.0	*KL*	−28.8	*PTPN13*	15.2
*CLK1*	12.5	*L3MBTL1*	20.1	*RBBP6*	−13.8
*CLK2*	−18.3	*LAMA4*	10.2	*RLN2*	−16.5
*COL13A1*	17.0	*LETMD1*	20.1	*RNF135*	−24.7
*COL8A1*	11.2	*LPPR5*	−23.2	*RUNX2*	−16.3
*CSPP1*	15.6	*MTMR3*	−13.3	*SLC3A2*	−18.8
*CTSB*	−24.5	*MYH11*	−11.8	*SYK*	16.5
*DCLRE1C*	−15.9	*MYO18A*	−16.1	*TM2D2*	−14.4
*DLEC1*	−53.8	*NAA60*	11.7	*TMTC4*	13.3
*DMBX1*	−13.8	*NDEL1*	−11.2	*TRPV4*	−18.3
*DTX2*	−11.0	*NFATC2*	18.5	*TSGA10*	14.9
*EFNA1*	−13.0	*NFU1*	−12.0	*TTC23*	−12.7
*EPS8L3*	13.8	*NRXN2*	−11.8	*ZNF493*	25.0
*FAM13B*	20.4	*NTRK2*	41.0	*ZRANB2*	−11.5
*FAM86A*	28.6	*ODF2L*	−13.1		

### Characterization of changes in AS

**Fig. 3:**
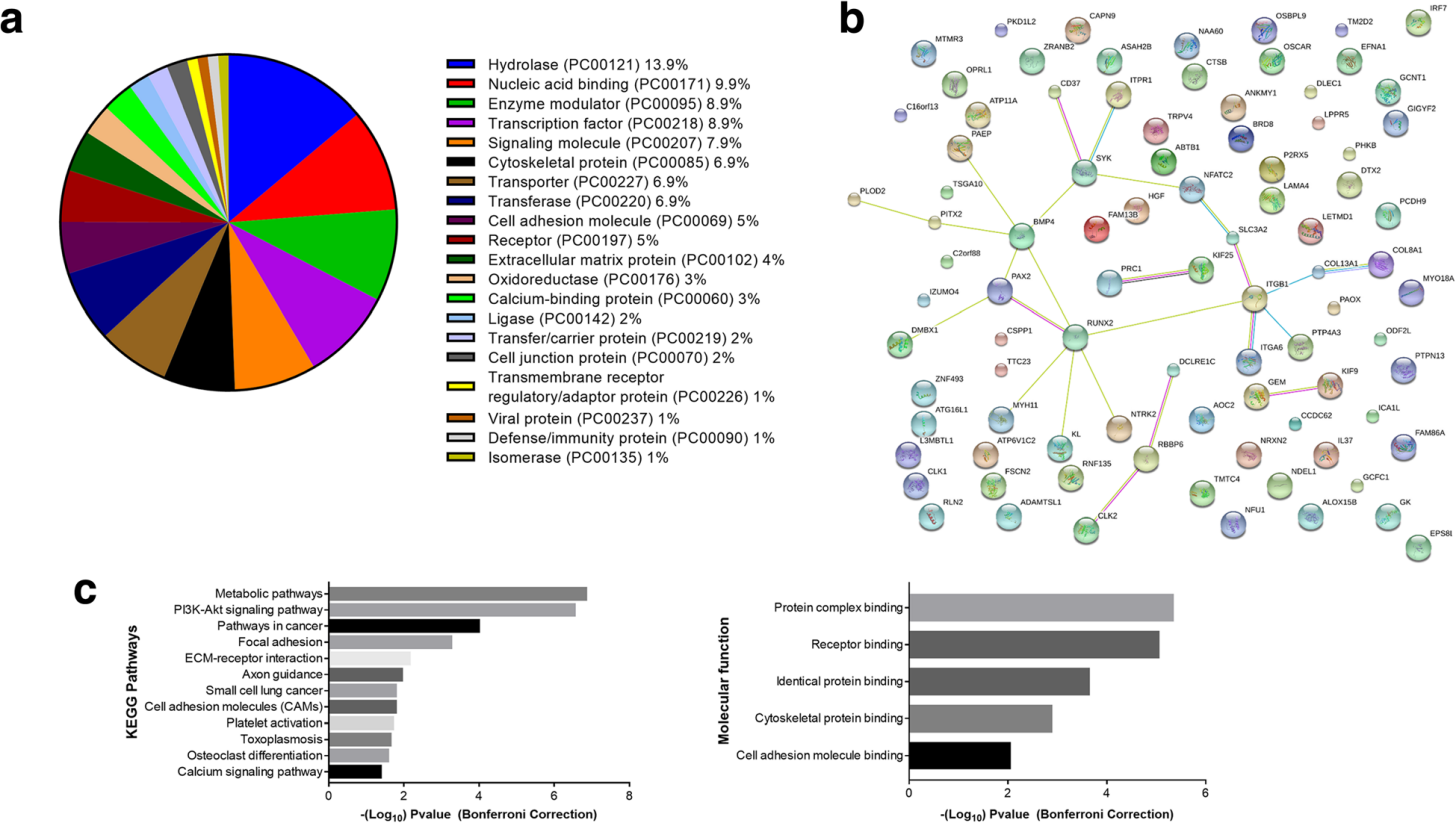
Bioinformatic analysis of genes that have their splicing changed in EBNA1-expressing cells; (**a**) Pie chart of the different protein class for the genes with EBNA1-driven change in splicing. A wide range of classes are present, showing that EBNA1 does not seem to impact the splicing of one particular class of protein; (**b**) String network of the 89 genes that have their splicing modified in EBNA1-expressing cells. A small network of interacting protein including SYK, BMP4, PAX2 and RUNX2 is present (*p* = 0.0324); (**c**) Gene Ontology analysis using DAVID reveals enrichment in KEGG pathways linked to adhesion, signaling and cancer mainly. Molecular function also reveals this enrichment, as seems by GO terms linked to receptor, cytoskeletal and cell adhesion binding, and cell metabolism

Genes for which the AS profiles were modified in EBNA1-expressing cells belong to numerous families and have widespread activities. For example, transcripts encoding ATP binding cassette transporters (*ATP11A*, *ATP6V1C2*), immune responses effectors (*IL37*, *IRF7*), transcription factors (*PAX2*, *ZNF493*), RNA splicing factors and their regulators (*CLK1*, *CLK2*, *ZRANB2*), proteases (*CAPN9*), and signaling proteins (*BMP4*, *CD37*) have their AS modified in EBNA1-expressing cells. Analysis of protein classes using PANTHER revealed the wide diversity of protein activity encoded by these genes, with hydrolase and nucleic acid binding being the most abundant ones (Fig. 3a). Next, the possibility that genes which have their splicing modulated by EBNA1 belongs to a specific network or pathway was assessed using STRING Network. Protein-protein interactions showed a small significant network (*p* = 0.03) involving RUNX2, PAX2, SYK, BMP4, and ITGB1 amongst others (Fig. 3b). Interestingly, many of these proteins are transcription factors or have known roles in morphogenesis and cellular differentiation. To determine if a specific biological role was targeted by EBNA1, gene ontology analysis was carried using DAVID for the KEGG pathways and molecular functions. As predicted, many pathways involved in cancers, such as the PI3K-AKT pathway, are enriched, since the assay focused on genes involved in cancer (Fig. 3c). Interestingly, many terms linked to adhesion, such as focal adhesion, ECM-receptor interaction, cell adhesion molecules (CAMs), and cell adhesion molecule binding are enriched, which could indicate preferential modulation of AS for genes involved in adhesion.

### Deciphering EBNA1 modulation of AS

**Fig. 4:**
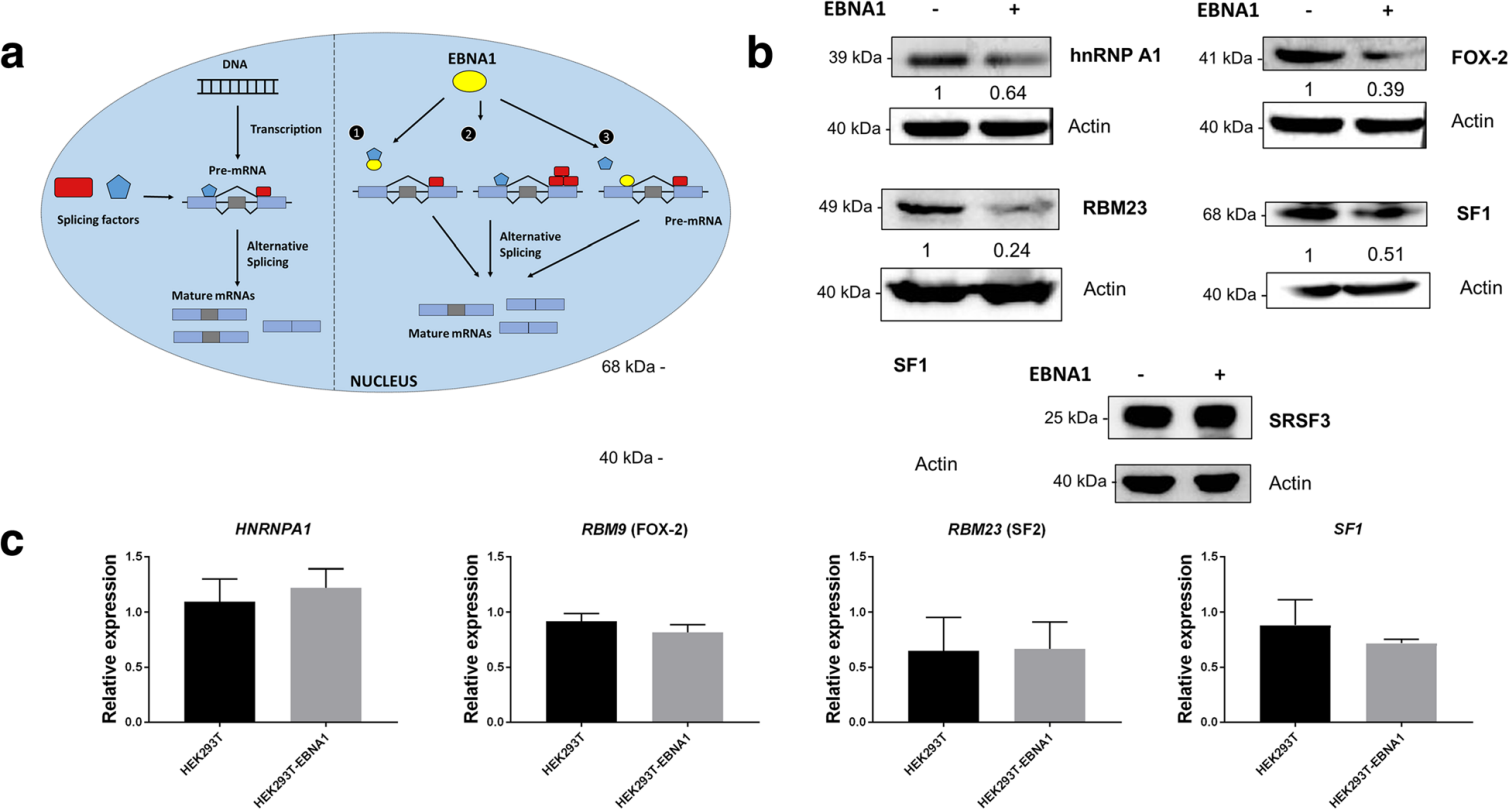
Investigation into the molecular mechanism responsible for the modulation of AS by EBNA1; (**a**) Diagram representing the AS pathway in the nucleus and how the EBNA1 protein could potentially impact this pathway. Mainly, EBNA1 could bind to splicing factors [1], thus impacting either their activity or their binding to nascent pre-mRNAs; EBNA1 could also lead to changes in the expression of splicing factors [2], thus disturbing the ratio of enhancing and silencing splicing factors; EBNA1 could also bind to nascent pre-mRNAs [3], thus preventing the binding of other splicing factors and/or acting itself as a splicing enhancer or inhibitor; (**b**) Western blot analysis of splicing factors protein levels shows that EBNA1 diminishes proteins levels of hnRNPA1, FOX-2, RBM23, and SF1. Actin was used as a loading control on the same membrane after stripping, and relative quantitation reported to the actin level is indicated underneath. A Western blot against SRSF3 is also included and EBNA1 does not affect its level, hence showing that the effect of EBNA1 is not widespread against splicing factors in general; (**c**) qPCR analysis of splicing factors shows no statistically significant difference between control and EBNA1-expressing cells at the mRNA level (*n* = 3 for each condition)

High-throughput studies reveal global portraits of what is happening in a particular condition/circumstance in a cell and thus can simultaneously reveal various molecular mechanisms leading to the global picture observed. Dissecting those mechanisms and their contributions to the observed outcomes is the primary challenge of this type of study. In the current case, many different mechanisms might explain how the EBNA1 protein is able to achieve modulation of AS, as outlined in Fig. 4a. First, EBNA1 could bind to splicing factors through direct protein-protein interaction, thus impacting either their activity and/or their binding to nascent pre-mRNAs. Second, EBNA1 expression could lead to a change in the expression of splicing factors, thus disturbing the ratio of enhancing and silencing splicing factors on pre-mRNA. Finally, EBNA1 could bind to nascent pre-mRNA in the nucleus, thus preventing the binding of splicing factors and/or acting itself as a splicing enhancer or inhibitor. It should be noted that EBNA1 interaction with splicing factors has been previously demonstrated as EBNA1 is able to bind to the hnRNPH1 splicing factor [28]. Since the first possibility has already been demonstrated, we focused on the latter two possibilities.

In a previous study, we had shown that in EBV-positive gastric carcinomas, several splicing factors had their expression changed compared to healthy tissues [28]. To study the possibility that EBNA1 disrupts the expression of splicing factors, protein levels of splicing factors (SF) were assessed in HEK293T and HEK293T-EBNA1 cells. We probed various SF from the hnRNP family (hnRNPA1 and hnRNPH1), the SR family (SRSF2, phosphorylated-SRSF2, SRSF3, SRSF6, SRSF9 and SRSF10), as well as general SF (ESRP1, FOX-2, RBM23, and SF1). Analysis of proteins level by Western blotting clearly showed that EBNA1 decreases the abundance of SF1, RBM23, hnRNPA1, and FOX-2 by 1.5 to 4-fold (Fig. 4b). However, the protein levels of hnRNPH1, p-SRSF2, SRSF3, SRSF6, and SRSF10 remained constant following EBNA1 expression. A representative Western blot for SRSF3 is shown in Fig. 4b. Note that Western blots against ESPR1, SRSF2 and SRSF9 were inconclusive. Gene expression levels for these splicing factors (SF1, RBM23, hnRNPA1, and FOX-2) were also quantified using qPCR and failed to detect any statistically significant transcriptional effect of EBNA1 on these genes (Fig. 4c), suggesting that the expression of EBNA1 can alter the expression of specific splicing factors at the protein level, either by affecting their translation or their stability.

### RIP-sequencing of EBNA1-bound RNA

**Fig. 5:**
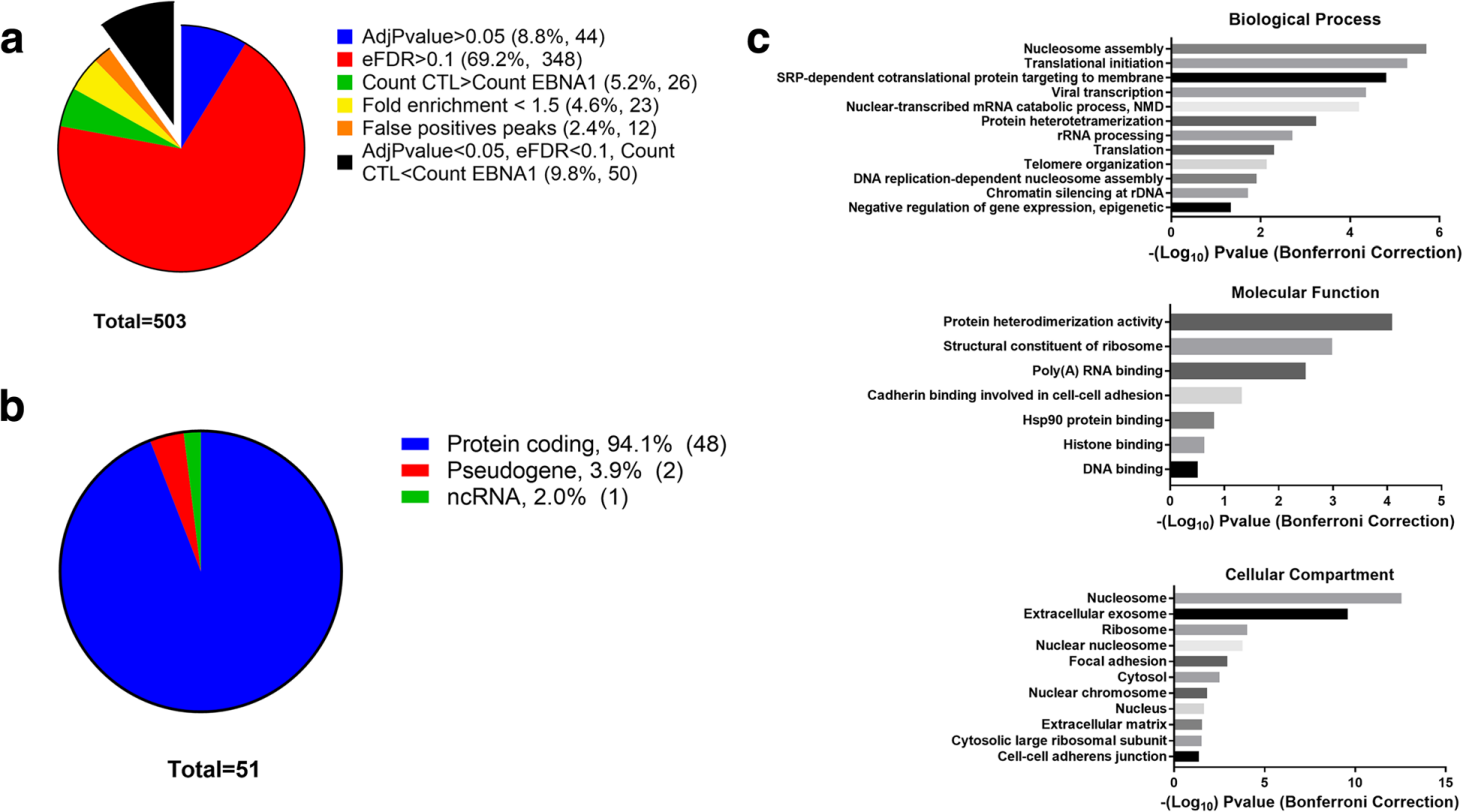
RIP-Sequencing of RNAs bound to the EBNA1 protein reveal that EBNA1 is bound to cellular RNAs in cellulo; (**a**) Statistical filtering used to select only high confidence peaks from the RIPSeeker analysis. To be selected, peaks needed to pass the following thresholds: Adjusted Pvalue lower than 0.05; False discovery rate lower than 10%; count in the EBNA1 analysis higher than the control; fold-enrichment higher than 1.5; manual curation do not show a false-positive peak. This allowed us to select only 50 peaks (black slice) from the 503 outputted by the analysis, from which one of the peaks was separated into two distinct peaks upon manual curation, for a total of 51; (**b**) Distribution of annotations corresponding to identified peaks from the ENSEMBL database. Annotations were retrieved using the online BioMart interface; (**c**) Gene ontology analysis for biological processes, cellular compartments and molecular functions of the 51 annotations retrieved from selected RIPSeeker peaks. The DAVID Bioinformatics Resources version 6.8 was used to retrieve enrichment, using the human genome as a control. A statistical threshold of less than 0.05 on the Benjamini corrected Pvalue was used

**Fig. 6:**
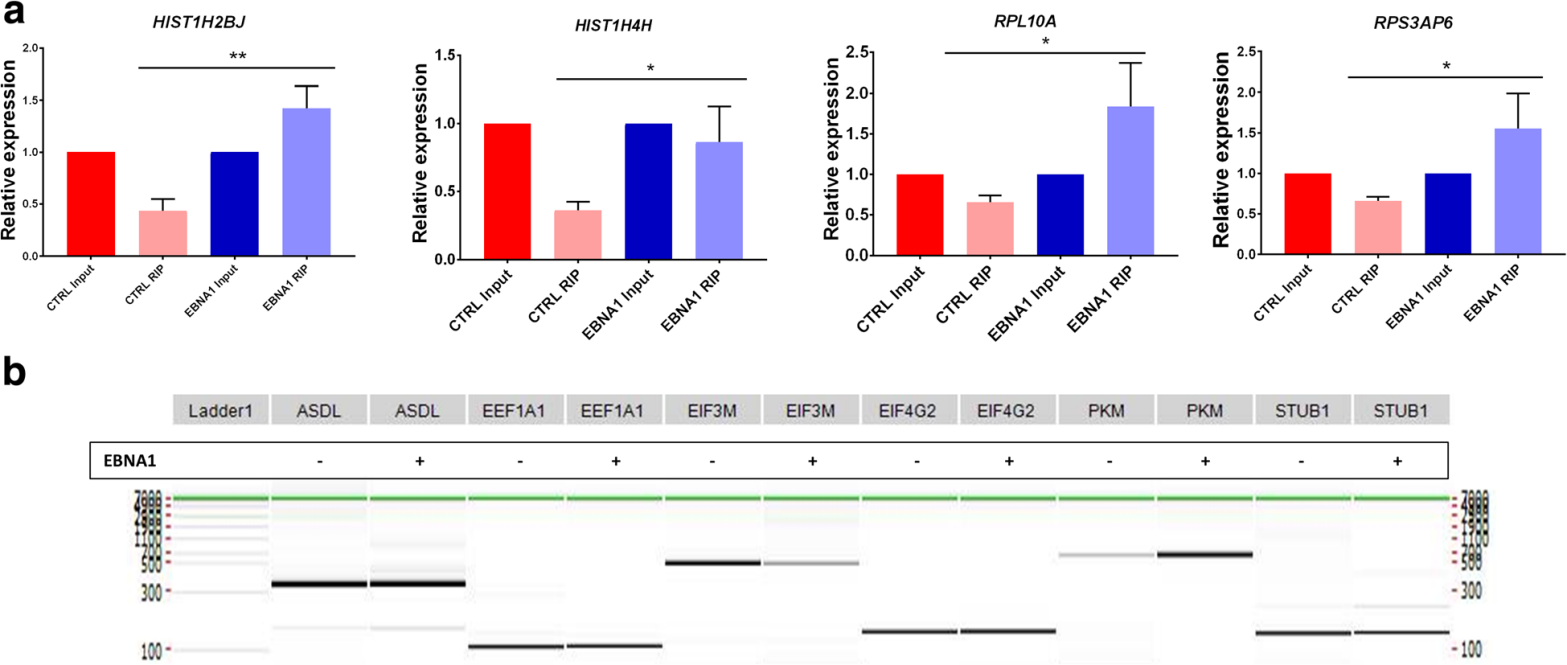
Binding of EBNA1 to cellular mRNAs in cellulo; (**a**) RIP-qPCR validation of RNA binding targets of the EBNA1 protein. RIP experiments from biological triplicates of HEK293T and HEK293T-EBNA1 cells were carried out, and immunoprecipitated RNAs were quantified in qPCR and normalized to their respective input RNA. HIST1H2BJ, HIST1H4H, RPL10A and RPS3AP6 were statistically enriched in EBNA1 immunoprecipitated fraction compared to control immunoprecipitation. Unpaired t-test, * *p* < 0.05, ** *p* < 0.005; (**b**) AS-PCR on potential AS site detected in the RIP-Seq experiment. Primers were designed to amplify ASEs potentially bound by EBNA1. Control and EBNA1 cells were compared to detect any change in AS in these regions. No significant change could be detected for any potential AS regions bound by EBNA1

The last possibility to explain EBNA1 modulation of AS involves EBNA1 binding directly to mRNAs. Interestingly, it was previously demonstrated that EBNA1 is able to bind RNA and RNA G-quadruplex (G4) structures (i.e. guanine-rich secondary structure formed of stacked guanine quartet) [35, 43, 44]. Moreover, the EBNA1 mRNA harbors such G4 structures, leading to the hypothesis that EBNA1 could bind its own mRNA [35]. This raised the possibility that EBNA1 might bind to nascent pre-mRNAs in the nucleus in a sequence- or structure-dependent manner, potentially altering the splicing of cellular transcripts. To seek out if EBNA1 was bound to mRNAs which have their splicing modulated, RIP-seq was carried out from both HEK293T and HEK293T cells expressing EBNA1. A flowchart summarizing the RIP-Seq protocol is outlined in Additional file 1: Figure S3. Numerous experimental controls were performed to ensure recovery of high-quality RNA for sequencing. First, immunoprecipitations were validated by Western-Blotting and showed efficient immunoprecipitation of the EBNA1 protein only in EBNA1-expressing cells (Additional file 1: Figure S4). Total lysates were also processed as IP fractions and then assessed on Agilent Nano Chip to evaluate RNA degradation through ribosomal 28 s/18 s ratio. Satisfactory RNA integrity numbers were obtained (9.2 for EBNA1 cells; 8.9 for control cells), hence revealing very limited RNA degradation resulting from the experimental procedure (Additional file 1: Figure S5). Immunoprecipitation fractions also showed strong peaks from the 28S and 18S ribosomal RNAs, probably due to carry over of highly abundant rRNA during the immunoprecipitation procedure (Additional file 1: Figure S5). To ensure appropriate depth of sequencing, those rRNAs were depleted using Illumina Ribo-Zero. Upon depletion, samples were completely free of rRNA, as seen from the results of the Agilent Nano Chip analysis (Additional file 1: Figure S6). Libraries were built and then quality was assessed; results are shown in Additional file 1: Figure S7. Upon sequencing, more that 20 million high quality reads were obtained from both conditions. To determine enriched peaks in the RIP data, reads were processed using a standard bioinformatic protocol (trimming using Trimmomatic, alignment using STAR on the hg38 genome, PCR and sequencing duplicates removal using samtools rmdup, peak calling with RIPSeeker). The analysis yielded 503 peaks corresponding to 527 unique annotations; results are available online with the raw reads (GEO Series accession number GSE107808). To ensure sufficient stringency, data were filtered using strict statistical thresholds: Benjamini-corrected *P*-values under 0.05; false discovery rate under 0.1; count numbers from the EBNA1 dataset higher than in the control dataset, and a fold-enrichment relative to the control greater that 1.5 (Fig. 5a). Further manual curation using the read coverage over these regions identified 12 false positive peaks, narrowing down the list of probable EBNA1-bound RNAs to 50. Manual curation also allowed to validate the real positioning of the peaks, and to further separate a peak overlapping histones *HIST1H2AC* and *HIST1H2BC* into two separates peaks. The complete list of manually curated peaks can be visualized in Table S3. Interestingly, the vast majority of these annotations correspond to protein-coding genes (94%), with the rest being two pseudogenes and one non-coding RNA (Fig. 5b). Gene ontology analysis using the DAVID bioinformatic database showed enrichment in biological processes linked to ribosome biogenesis, translation, and chromatin organization (Fig. 5c, Bonferroni adjusted *p*-value< 0.05). Cellular compartment and molecular function analysis confirmed enrichment in EBNA1 bound transcripts for genes encoding proteins from the nucleosome and ribosome and with functions such as structural constituent of ribosome and poly(A) RNA binding. Interestingly, the term adhesion is also enriched as previously seen in EBNA1 modulation of AS (Fig. 3). This might suggest that EBNA1 could target this pathway both through AS modulation and direct RNA binding. To further validate these results, RIP experiments were carried out in biological triplicates from HEK293T and HEK293T-EBNA1 cells, and levels of immunoprecipitated RNAs were quantified using qPCR. Each immunoprecipitation was normalized to its input RNA, and relative expression from EBNA1-expressing cells and control cells were compared for 11 EBNA1 interactors as predicted by our RIP-Seq experiment. Levels of immunoprecipitated RNAs were statistically higher when EBNA1 was present for *HIST1H2BJ*, *HIST1H4H*, *RPL10A*, and *RPS3AP6* (Fig. 6a), hence showing that EBNA1 is able to interact with cellular RNAs such as mRNA (*HIST1H2BJ*, *HIST1H4H*, *RPL10A*) and non-coding RNA (*RPS3AP6*). It was not possible to prove a conclusive statistical enrichment for the 7 other predicted EBNA1 targets as predicted by the RIP-Seq. However, none of the EBNA1-bound targets predicted by RIP-Seq belonged to identified genes that had their splicing modulated following EBNA1 expression (Table 1 and Additional file 1: Table S3). Since we only focused previously on approximatively 1500 ASEs, one possibility would be that the transcripts bound by EBNA1 were not part of our initial AS screening. To assess the possibility that EBNA1-bound transcripts have their splicing modulated through EBNA1 interaction, the 51 regions of interaction determined by RIP-Seq were analyzed for potential ASE regions. Three of these regions covered an exon cassette and 3 others spanned the intron between two exons (possible intron retention) (Table S2). RT-PCR designed on these potential ASEs bound by EBNA1 failed to validate any change in the splicing outcome in these regions (Fig. 6b). Therefore, these findings suggest that EBNA1 does not modulate AS through direct binding to cellular mRNAs but provide clear evidence that EBNA1 binds to cellular mRNAs *in cellulo*.

## Discussion

Previous studies had already shown that EBV can disturb the splicing of cellular genes using few different mechanisms. For instance, the viral SM protein acts as a splicing factor, recruits SRSF3 to modulate splicing and competes with SRSF1 for RNA-binding. The only known gene to have its splicing impacted by SM is *STAT1*, for which SM is able to favor the STAT1β isoform, which is a dominant negative suppressor of STAT1α [17, 18]. Moreover, EBV expresses EBER1 and EBER2, two long non-coding RNAs accumulating in the nucleus during latent infection. EBER1 was not demonstrated to impact the splicing of cellular genes, but it is likely since it interacts with AUF1/hnRNP D splicing factor [19]. Expression of both EBERs in cells lead to significant changes in the expression and the splicing of cellular genes, but the effect of EBER1 and EBER2 alone were not investigated [20]. The current study is the first to look at modifications of AS using a high-throughput approach aimed at understanding how a single EBV molecule (protein or RNA) is impacting the AS patterns in the host cell. However, such experiments should ultimately be repeated in different cellular contexts, such as B cells and epithelial cells, to have a better understanding of the changes that are cell-type specific from those that are ubiquitous. Such studies would also give significant insight into the mechanistic behind these changes, and a more precise role in EBV infection. Moreover, this paper focused on genes involved in cancer, but a broader approach looking at the whole cellular AS using RNA-Seq, as it was recently used in the context of viral infection, could have identified more ASEs modulated by EBNA1, which is one of the drawback of the current approach [9, 10]. Nevertheless, although our results are limited, they establish sufficient evidences to justify follow-up studies using high-throughput sequencing technique, knock-down assays to confirm a reverse phenotype, and expression of EBNA1 in additional cell lines to further validate these findings.

Some of the genes with splicing change in EBNA1-expressing cells are particularly interesting in regards to viral carcinogenesis and replication. For example, the calpain CAPN9 was showed to be downregulated in gastric cancer [45, 46], leading to the hypothesis that this protein may act as a tumor suppressor. EBNA1 modulation of *CAPN9* splicing (ΔPSI = 19) could impact the level of the expressed protein or its isoform ratio. Since EBV is known to be implicated in gastric cancer, splicing modification could be another way of diminishing the level of CAPN9 protein, but at the post-transcriptional level. Further studies in this field have great potential regarding a better understanding of viral carcinogenesis. On the other hand, EBNA1 also modulates the splicing of genes previously known to have potential implication in its replicating cycle. The binding of IRF7, a known key player of antiviral immunity, to the BamHI Q promoter (Qp) of EBV was previously demonstrated [47]. This interaction represses transcription of EBNA1 gene downstream of Qp. However, the Qp promoter is only used in type-1 latency, which is characterized by exclusive expression of EBNA1 and low levels of IRF7 protein. IRF7 is upregulated by another EBV protein, LMP-1 during type III latency, which leads to the IRF7 inactivation of Qp. In this type of latency, other promoters are used to transcribe EBNA1. This led to the hypothesis that IRF7 might be involved in the regulation of EBV latency. EBNA1 modification of *IRF7* splicing (ΔPSI = 13) could be another way of regulating EBV promoter usage through IRF-7 post-transcriptional modulation.

The current study also investigated the nature of the cellular RNAs bound to EBNA1 using high-throughput sequencing. Our analysis showed a relatively low number of cellular RNAs potentially bound by EBNA1. This is in part due to the high stringency of statistical filters used to select data. It might also be attributable to the background noise of the experiment and the transcriptional effect of the EBNA1 protein, i.e. some genes seemed to be over-expressed in EBNA1-expressing cells, hence leading potentially to more non-specifically immunoprecipitated RNA transcripts. Nevertheless, we validated four different RNAs enriched during EBNA1 immunoprecipitation (Fig. 6a) that belong mainly to enriched GO terms from the RIP-Seq analysis, i.e. genes that are either histones or ribosomal proteins (Fig. 5c), demonstrating that the RIP-Seq data are representative of the biological role of EBNA1 in binding to cellular RNAs. Interestingly, the enrichment of these RNAs in EBNA1 RIP are relatively low. This finding might suggest that the binding of EBNA1 to cellular RNA is either transitory or of low affinity. An interesting hypothesis would be that a tight binding of EBNA1 to cellular RNAs would likely sequester the protein away from its main role in replication and segregation of the EBV episome during latency, and therefore be deleterious for viral latency [36, 44]. It will be interesting to study the role of EBNA1 binding to cellular RNAs when EBNA1 is not needed for the maintenance of the viral episome (i.e. not during cellular division). Since the potential binding of EBNA1 to its own mRNA was previously suggested [35, 48], we also looked at the RIP-Seq data to question if such binding occurs in cells. Our result indicated that nearly 7000 reads originated from the EBNA1 mRNA in the HEK293T-EBNA1, and no read was detectable in the control HEK293T (Additional file 1: Figure S8). This suggests that EBNA1 interacts with its own mRNA in a cellular context. A previous study suggested that EBNA1 could potentially bind to G-quadruplex (G4) located in the coding sequence for the glycine-alanine repeats (GAR) domain, i.e. between nucleotides 270 and 984 of the EBNA1 coding sequence [35]. To verify if EBNA1 indeed pulled down a specific region of its own mRNA, the same approach as previously used to validate EBNA1-bound mRNA (RIP-qPCR) was used. Primers were designed to amplify regions in the beginning (37–131), the middle (1137–1278) and the end (1752–1837) of the EBNA1 coding sequence (shown in red in Additional file 1: Figure S8). However, this experiment failed to validate an enrichment in any region of the coding sequence (Additional file 1: Figure S8). Clearly, further studies will be needed to have a clear understanding of the binding of EBNA1 to its own mRNA. The investigation of the dynamics of EBNA1 interaction with cellular RNAs and with its own mRNA will be interesting to better understand the impact of EBNA1 in EBV-positive latent cells.

Surprisingly, EBNA1-bound RNAs are not spliced differentially, either by comparing the genes determined by high throughput RT-PCR (Table 1) with the RIP-Seq peaks (Table S3) or by doing AS-PCR on ASE regions where EBNA1 is predicted to bind by RIP-Seq (Fig. 6b). As stated in Fig. 4a (third hypothesis), modulation of AS through direct interaction with cellular RNAs was a possible manner by which EBNA1 could potentially change the AS of cellular genes. However, our results do not support this hypothesis, and point toward the two other proposed mechanisms (modulation of expression of splicing factors and interaction with splicing factors) as the ones used by EBNA1 to modulate AS (Fig. 4a). The ability of EBNA1 to modulate the expression of splicing factors such as SF1, SF2, hnRNPA1 and FOX-2 (Figs. 4b and c) corroborates the second hypothesis. However, differential motif enrichment analyses using DREME [49] on the sequences located between the primer pairs used for RT-PCR, as compared to 89 unchanged ASEs randomly selected, yielded no significant enrichment in a sequence that could be linked to the changes in AS. Indeed, it is probable that the sum of all theses changes in expression of SF is leading to the change in alternative splicing, and thus for every ASEs that we have analysed there is a specific combination of some of the splicing factors that have their expression changed that are responsible for the change in AS. This probably explain why no specific motif could be identified by enrichment analysis. As previously demonstrated, EBNA1 interacts with splicing factor hnRNPH1 [28] (first hypothesis). Further studies will be needed to decipher the role of changes in splicing factor expression and direct interaction with splicing factors on the AS of cellular gene and how the EBNA1 protein exploits these characteristics during EBV infection, latency and carcinogenesis.

Both in the context of viral infection and carcinogenesis, it is tempting to speculate that the development of splice-switching oligonucleotides specific to virally-induced splicing modifications could help both the prevention and the treatment against diseases arising from viral infection. The development of such tools is already accelerating, and the identification of splicing events modulated by viruses that could have impact on viral replication or carcinogenesis is likely the first step towards their usage in this context.

## Conclusion

This study demonstrates that the expression of the EBV EBNA1 protein in cells induces changes in the AS pattern of numerous cellular genes previously shown or suspected to be implicated in cancer. These modifications could have drastic implication in viral carcinogenesis and EBV latency, since they are likely to change the proteome diversity in EBNA1-expressing cells. Overall, these data pave the way to a better understanding of virus-host interaction, latency of herpesviruses, viral carcinogenesis, and the implication of splicing in these phenomena.

## Additional files

Additional file 1: The following are available online at www.mdpi.com/xxx/s1, **Table S1** List of gene names and their respective official full names **Table S2** AS-PCR primers used to analyze the AS of potential ASEs bound by EBNA1 **Table S3** List of the 51 peaks identified in EBNA1 RIP-Seq **Figure S1** Supplemental electrophoregrams of genes that have their splicing modulated upon EBNA1 expression, **Figure S2** Effect of transient MSCV-N transfection on cellular AS. **Figure S3** Flowchart summarizing the RIP-Seq protocol and controls used, **Figure S4** Western-Blotting of immunoprecipitation of EBNA1, **Figure S5** Quality assessment of input RNA and immunoprecipitated RNA for the RIP-Seq experiment, **Figure S6** Quality assessment of control and EBNA1 RIP following ribo-depletion, **Figure S7** Quality assessment of library for the RIP-Seq, **Figure S8** Read distribution on the EBNA1 coding sequence in the EBNA1 RIP-Seq and qPCR measurement of immunoprecipitatedEBNA1 mRNA. (PDF 2818 kb)
