# Supplementary material for: The Epstein-Barr virus EBNA1 protein modulates the alternative splicing of cellular genes
Source: Virol J. 2019 Mar 4;16:29. doi: 10.1186/s12985-019-1137-5 (PMC6399920; doi:10.1186/s12985-019-1137-5)
Supplement: Supplementary file 1 — The following are available online at www.mdpi.com/xxx/s1, Table S1 List of gene names and their respective official full names Table S2 AS-PCR primers used to analyze the AS of potential ASEs bound by EBNA1 Table S3 List of the 51 peaks identified in EBNA1 RIP-Seq Figure S1 Supplemental electrophoregrams of genes that have their splicing modulated upon EBNA1 expression, Figure S2 Effect of transient MSCV-N transfection on cellular AS. Figure S3 Flowchart summarizing the RIP-Seq protocol and controls used, Figure S4 Western-Blotting of immunoprecipitation of EBNA1, Figure S5 Quality assessment of input RNA and immunoprecipitated RNA for the RIP-Seq experiment, Figure S6 Quality assessment of control and EBNA1 RIP following ribo-depletion, Figure S7 Quality assessment of library for the RIP-Seq, Figure S8 Read distribution on the EBNA1 coding sequence in the EBNA1 RIP-Seq and qPCR measurement of immunoprecipitatedEBNA1 mRNA. (PDF 2818 kb) [file 12985_2019_1137_MOESM1_ESM.pdf]

## Supplementary Data

# The Epstein-Barr virus EBNA1 protein modulates the alternative splicing of cellular genes

Simon Boudreault[1], Victoria E. S. Armero[1], Michelle S. Scott[1], Jean-Pierre Perreault[1], Martin Bisaillon[1*]

| Official Symbol | Official Full Name |
|---|---|
| *ATP11A* | ATPase phospholipid transporting 11A |
| *ATP6V1C2* | ATPase H+ transporting V1 subunit C2 |
| *BMP4* | Bone morphogenetic protein 4 |
| *CAPN9* | Calpain 9 |
| *CCDC62* | Coiled-coil domain containing 62 |
| *CD37* | CD37 molecule |
| *CLK1* | CDC like kinase 1 |
| *CLK2* | CDC like kinase 2 |
| *DST* | Dystonin |
| *FAS* | Fas cell surface death receptor |
| *HIST1H2AC* | Histone cluster 1 H2A family member c |
| *HIST1H2BC* | Histone cluster 1 H2B family member c |
| *HIST1H2BJ* | Histone cluster 1 H2B family member j |
| *HIST1H4H* | Histone cluster 1 H4 family member h |
| *IL37* | Interleukin 37 |
| *IRF7* | Interferon regulatory factor 7 |
| *ITGB1* | Integrin subunit beta 1 |
| *LPPR5* | Phospholipid phosphatase related 5 |
| *NTKR2* | Neurotrophic receptor tyrosine kinase 2 |
| *PAX2* | Paired box 2 |
| *PCBP2* | Poly(rC) binding protein 2 |
| *RPL10A* | Ribosomal protein L10a |
| *RPS3AP6* | Ribosomal protein S3A pseudogene 6 |
| *RUNX2* | Runt related transcription factor 2 |
| *SYK* | Spleen associated tyrosine kinase |
| *ZNF493* | Zinc finger protein 493 |
| *ZRANB2* | Zinc finger RANBP2-type containing 2 |

Table S1: List of gene names and official full names

| Gene | ASE | Foward primer | Reverse primer | Short amplicon | Long amplicon |
|---|---|---|---|---|---|
| ASDL | Exon cassette | CATTTCTGAAGGATTGGTCGTG | CAGGGGATACACCTCCTCTTC | 157 | 334 |
| EEF1A1 | Possible intron retention | CAACATGCTGGAGCCAAGTGC | GCAGTCCAGAGCCTCAAGCAGC | 106 | 189 |
| EIF3M | Exon cassette | GCCTTCATCGACATCAGTGAAG | CTCTTCTTACAATCCACAAGTGCCTC | 125, 258, 387 | 487, 521, 599 |
| EIF4G2 | Possible intron retention | GGACAAAGCCCTAGAAGAGCC | CAAATTAGGAGGCGTCTGAATG | 150 | 233 |
| PKM | Exon cassette | GGGACTGCCTTCATTCAGACC | CAGCAGGCAAGTCCACAGCAG | 142 | 415, 545, 637 |
| STUB1 | Possible intron retention | CCAGTGGCATCACCTACGAC | GATGAATGCGTCAATAACCTCC | 143 | 225 |

Table S2: AS-PCR primers used to analyze the AS of potential ASEs bound by EBNA1

| Chromosome | Start | End | Width | Gene name | Count | Count CTL | PValAdj | eFDR | Fold enrichment |
|---|---|---|---|---|---|---|---|---|---|
| 11 | 106077801 | 106078000 | 200 | AASDHPPT | 430 | 64 | 4,6E-146 | 0,100 | 6,7 |
| 11 | 106090601 | 106090800 | 200 | AASDHPPT | 447 | 182 | 8,1E-144 | 0,086 | 2,5 |
| 22 | 40363001 | 40363200 | 200 | ADSL | 615 | 125 | 3,9E-219 | 0,100 | 4,9 |
| 22 | 40364801 | 40365000 | 200 | ADSL | 630 | 77 | 4,2E-304 | 0,000 | 8,2 |
| 11 | 112082601 | 112083000 | 400 | C11orf57 | 1426 | 409 | 1,1E-145 | 0,097 | 3,5 |
| 11 | 95813001 | 95813200 | 200 | CEP57 | 409 | 139 | 4,0E-138 | 0,080 | 2,9 |
| 7 | 133035366 | 133035570 | 205 | LOC729998 | 1446 | 43 | 0,0E+00 | 0,000 | 33,6 |
| 11 | 90211201 | 90211400 | 200 | CHORDC1 | 449 | 227 | 3,7E-142 | 0,080 | 2,0 |
| 6 | 73518751 | 73518975 | 225 | EEF1A1 | 2644 | 1589 | 0,0E+00 | 0,000 | 1,7 |
| 11 | 62573801 | 62574000 | 200 | EEF1G | 900 | 298 | 3,8E-140 | 0,075 | 3,0 |
| 11 | 32589001 | 32589200 | 200 | EIF3M | 426 | 156 | 6,0E-141 | 0,078 | 2,7 |
| 11 | 92354401 | 92354600 | 200 | FAT3 | 409 | 47 | 1,7E-142 | 0,081 | 8,7 |
| 11 | 110462801 | 110463000 | 200 | FDX1 | 406 | 68 | 8,9E-141 | 0,076 | 6,0 |
| 11 | 67585201 | 67585400 | 200 | GSTP1 | 421 | 159 | 8,7E-140 | 0,074 | 2,6 |
| 6 | 26156251 | 26156475 | 225 | HIST1H1E | 2452 | 555 | 0,0E+00 | 0,000 | 4,4 |
| 6 | 26123626 | 26124525 | 900 | HIST1H2AC | 12128 | 4705 | 0,0E+00 | 0,000 | 2,6 |
| 6 | 27132976 | 27133425 | 450 | HIST1H2AG | 6040 | 3027 | 0,0E+00 | 0,000 | 2,0 |
| 6 | 27147151 | 27147600 | 450 | HIST1H2AH | 5682 | 2481 | 0,0E+00 | 0,000 | 2,3 |
| 6 | 26123626 | 26124525 | 900 | HIST1H2BC | 12128 | 4705 | 0,0E+00 | 0,000 | 2,6 |
| 6 | 27132301 | 27132525 | 225 | HIST1H2BJ | 3680 | 743 | 0,0E+00 | 0,000 | 5,0 |
| 6 | 27146476 | 27146700 | 225 | HIST1H2BK | 3697 | 2157 | 0,0E+00 | 0,000 | 1,7 |
| 6 | 26031601 | 26031825 | 225 | HIST1H3B | 2437 | 1183 | 0,0E+00 | 0,000 | 2,1 |
| 6 | 27810001 | 27810225 | 225 | HIST1H3H | 3043 | 983 | 0,0E+00 | 0,000 | 3,1 |
| 6 | 26285401 | 26285625 | 225 | HIST1H4H | 3014 | 1360 | 0,0E+00 | 0,000 | 2,2 |
| 6 | 27823951 | 27824175 | 225 | HIST1H4J | 2626 | 1323 | 0,0E+00 | 0,000 | 2,0 |
| 11 | 108474601 | 108475000 | 400 | KDELC2 | 812 | 287 | 1,4E-137 | 0,078 | 2,8 |
| 11 | 18407201 | 18407600 | 400 | LDHA | 1669 | 1064 | 9,0E-132 | 0,073 | 1,6 |
| 6 | 27688051 | 27688275 | 225 | TRI-AAT2-1 | 3009 | 668 | 0,0E+00 | 0,000 | 4,5 |
| 11 | 106010601 | 106010800 | 200 | MSANTD4 | 446 | 184 | 1,5E-143 | 0,085 | 2,4 |
| 22 | 42090601 | 42090800 | 200 | NDUFA6 | 580 | 47 | 0,0E+00 | 0,000 | 12,3 |

Table S3: List of the 51 peaks identified in EBNA1 RIP-Seq

| Chromosome | Start | End | Width | Gene name | Count | Count CTL | PValAdj | eFDR | Fold enrichment |
|---|---|---|---|---|---|---|---|---|---|
| 15 | 72208576 | 72208800 | 225 | PKM | 1859 | 1089 | 0,0E+00 | 0,000 | 1,7 |
| 15 | 72209701 | 72209925 | 225 | PKM | 2944 | 993 | 0,0E+00 | 0,000 | 3,0 |
| 11 | 112233401 | 112233600 | 200 | PTS | 441 | 118 | 1,2E-145 | 0,096 | 3,7 |
| 11 | 110233201 | 110233400 | 200 | RDX | 438 | 121 | 6,4E-145 | 0,092 | 3,6 |
| 6 | 35469451 | 35469675 | 225 | RPL10A | 3615 | 1090 | 0,0E+00 | 0,000 | 3,3 |
| 22 | 39313601 | 39313800 | 200 | RPL3 | 826 | 148 | 5,4E-209 | 0,091 | 5,6 |
| 15 | 66501676 | 66501900 | 225 | RPL4 | 2525 | 1490 | 0,0E+00 | 0,000 | 1,7 |
| 15 | 66502576 | 66502800 | 225 | RPL4 | 1967 | 1247 | 0,0E+00 | 0,000 | 1,6 |
| 15 | 66503251 | 66503475 | 225 | RPL4 | 1800 | 1046 | 0,0E+00 | 0,000 | 1,7 |
| 15 | 69452776 | 69453000 | 225 | RPLP1 | 2692 | 1013 | 0,0E+00 | 0,000 | 2,7 |
| 6 | 34421626 | 34421850 | 225 | RPS10 | 3169 | 1390 | 0,0E+00 | 0,000 | 2,3 |
| 6 | 34424551 | 34424775 | 225 | RPS10 | 2769 | 856 | 0,0E+00 | 0,000 | 3,2 |
| 6 | 33271876 | 33272100 | 225 | RPS18 | 3207 | 488 | 0,0E+00 | 0,000 | 6,6 |
| 11 | 75404801 | 75405000 | 200 | RPS3 | 823 | 431 | 2,6E-143 | 0,084 | 1,9 |
| 11 | 75405601 | 75405800 | 200 | RPS3 | 899 | 555 | 3,3E-129 | 0,082 | 1,6 |
| 11 | 4133401 | 4133600 | 200 | RRM1 | 450 | 240 | 9,4E-142 | 0,080 | 1,9 |
| 7 | 22510436 | 22510640 | 205 | EEF1A1P6 | 2392 | 10 | 0,0E+00 | 0,000 | 239,2 |
| 16 | 682241 | 682445 | 205 | STUB1 | 859 | 95 | 8,6E-288 | 0,000 | 9,0 |
| 11 | 112085201 | 112085400 | 200 | TIMM8B | 441 | 268 | 7,0E-139 | 0,082 | 1,6 |
| 11 | 102397201 | 102398000 | 800 | TMEM123 | 2068 | 1156 | 4,7E-143 | 0,083 | 1,8 |
| 15 | 77046526 | 77046750 | 225 | TSPAN3 | 1769 | 513 | 0,0E+00 | 0,000 | 3,4 |

Table S3 (cont'd): List of the 51 peaks identified in EBNA1 RIP-Seq

Figure S1. Supplemental electrophoregram of genes that have their splicing modulated upon EBNA1 expression.

Total RNA was extracted from control HEK293T cells and EBNA1-expressing HEK293T cells, reverse transcribed and alternative splicing was assessed using primers designed to amplify only one ASE. Endpoint PCR products were resolved using capillary electrophoresis and detected using fluorescence. Red arrows indicate the short form for the ASE analyzed; black arrows indicate the long one.

# ALOX15B (ΔPSI=76.1)

HEK293T

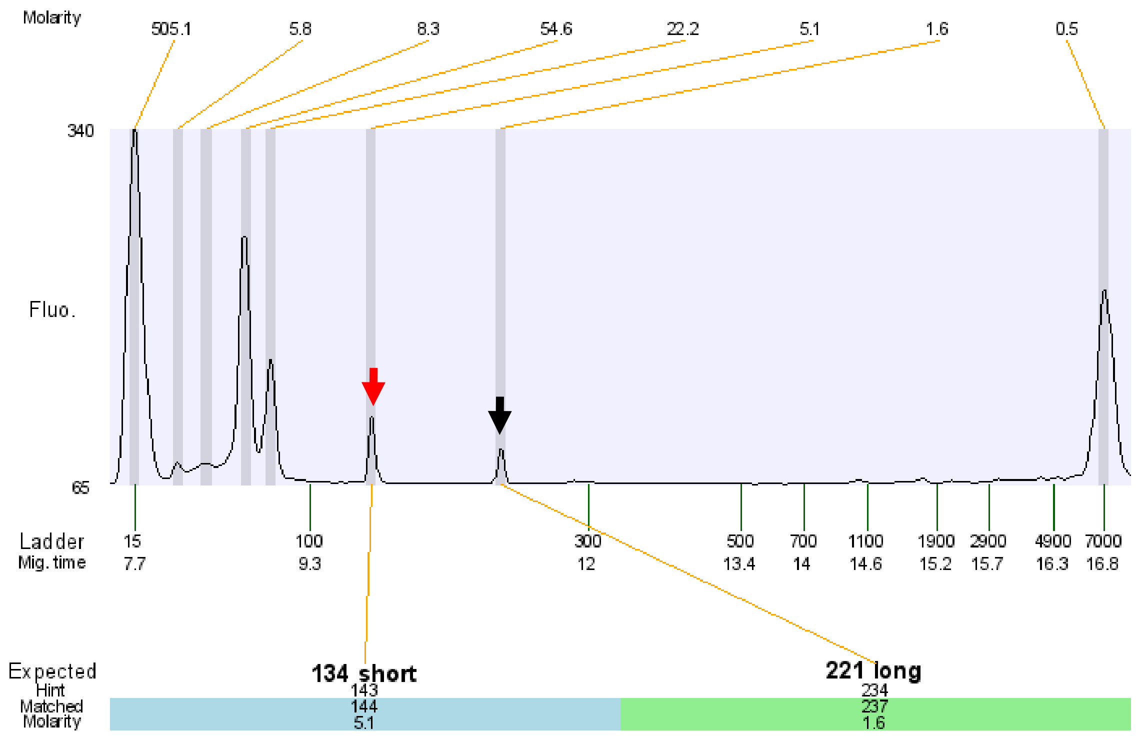


HEK293T-EBNA1

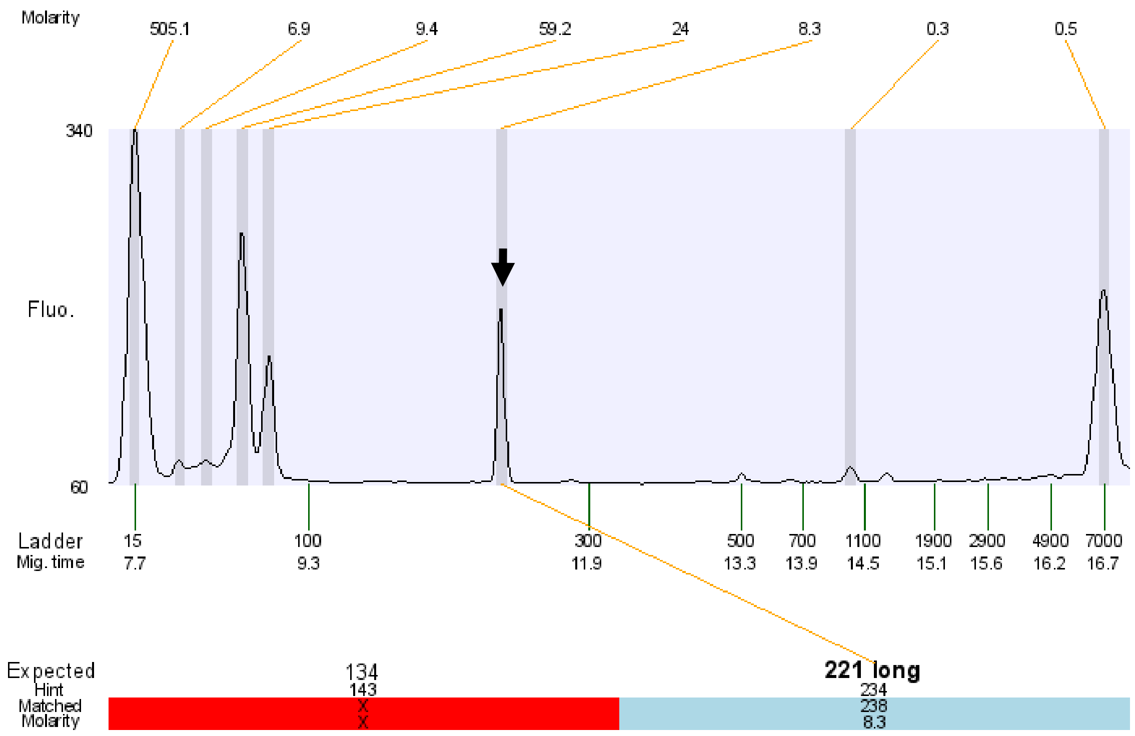


Figure S1

# BRD8 (ΔPSI=-20.1)

HEK293T

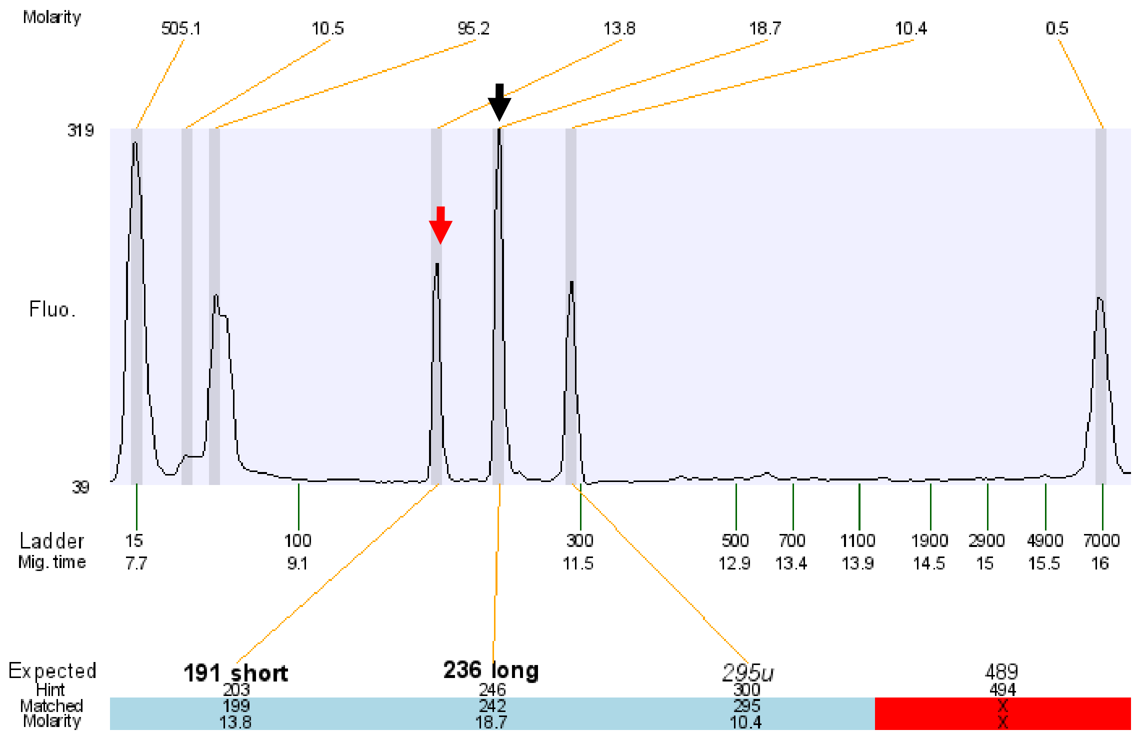


HEK293T-EBNA1

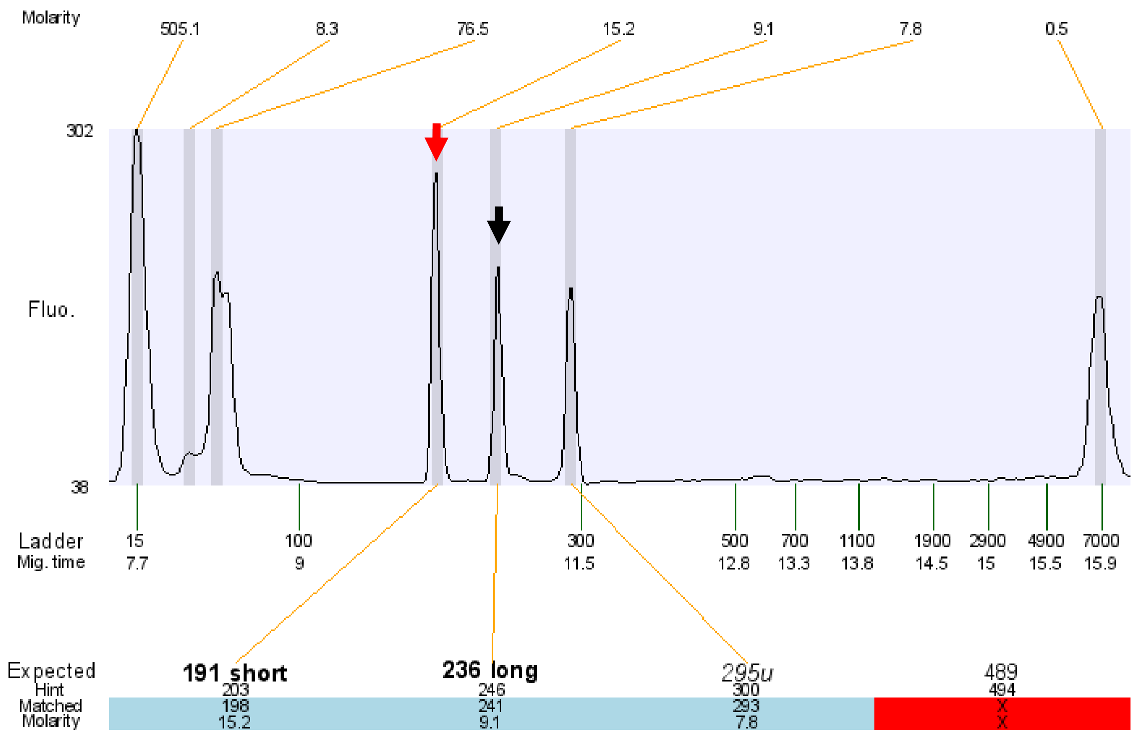


Figure S1 (cont'd)

# CAPN9 (ΔPSI=19)

HEK293T

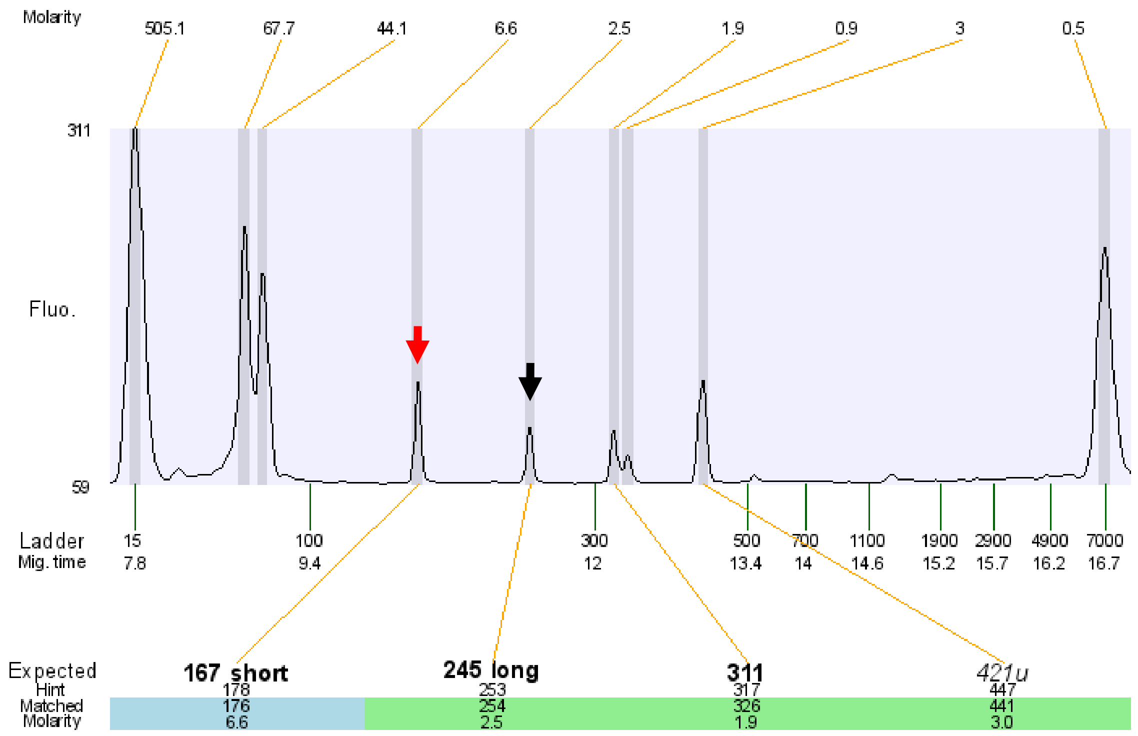


HEK293T-EBNA1

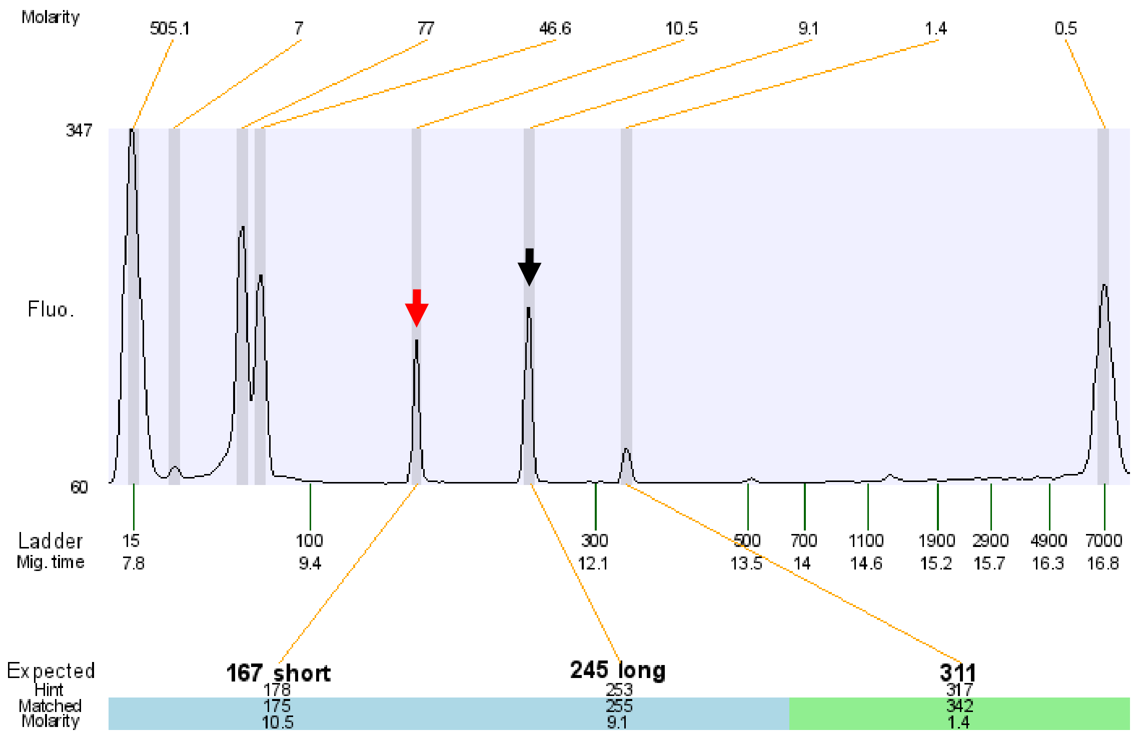


Figure S1 (cont'd)

# DLEC1 (ΔPSI=-53.8)

HEK293T

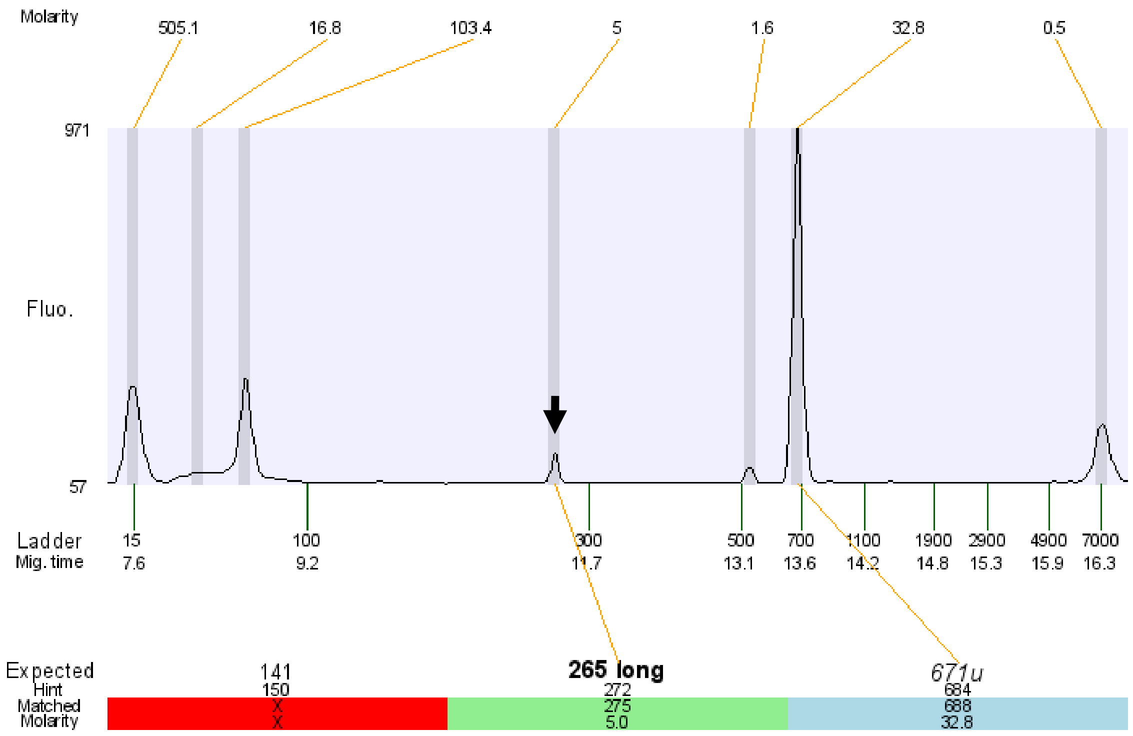


HEK293T-EBNA1

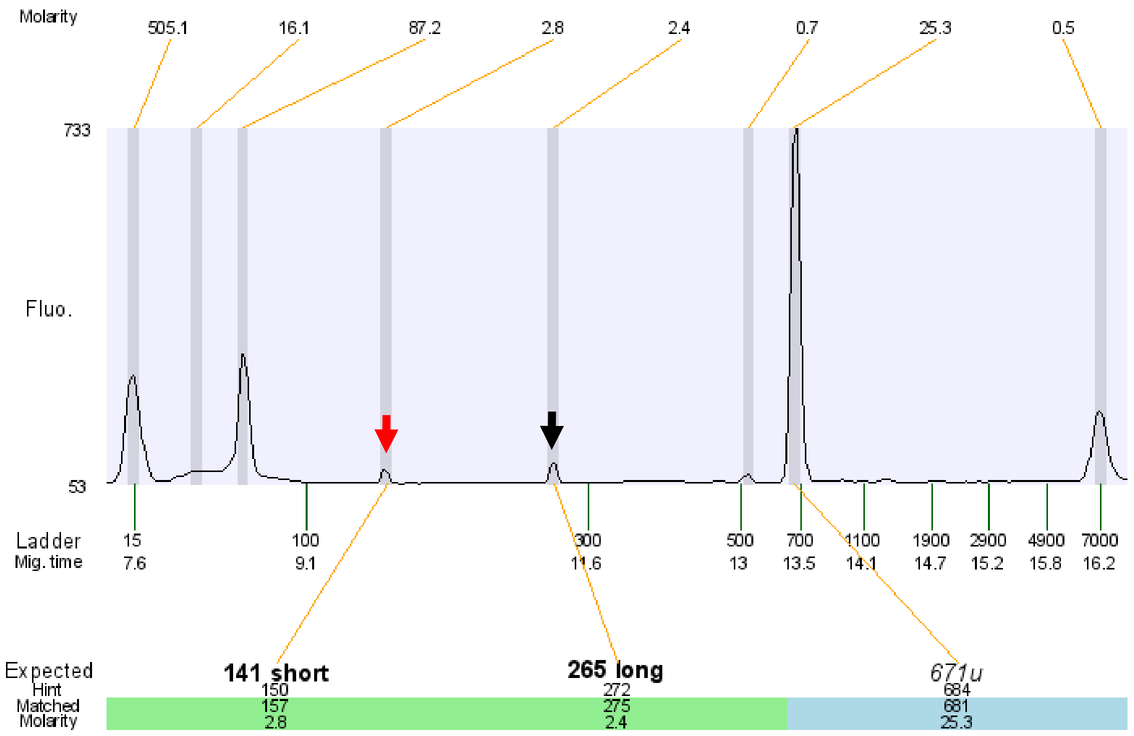


Figure S1 (cont’d)

# HGF (ΔPSI=24.4)

HEK293T

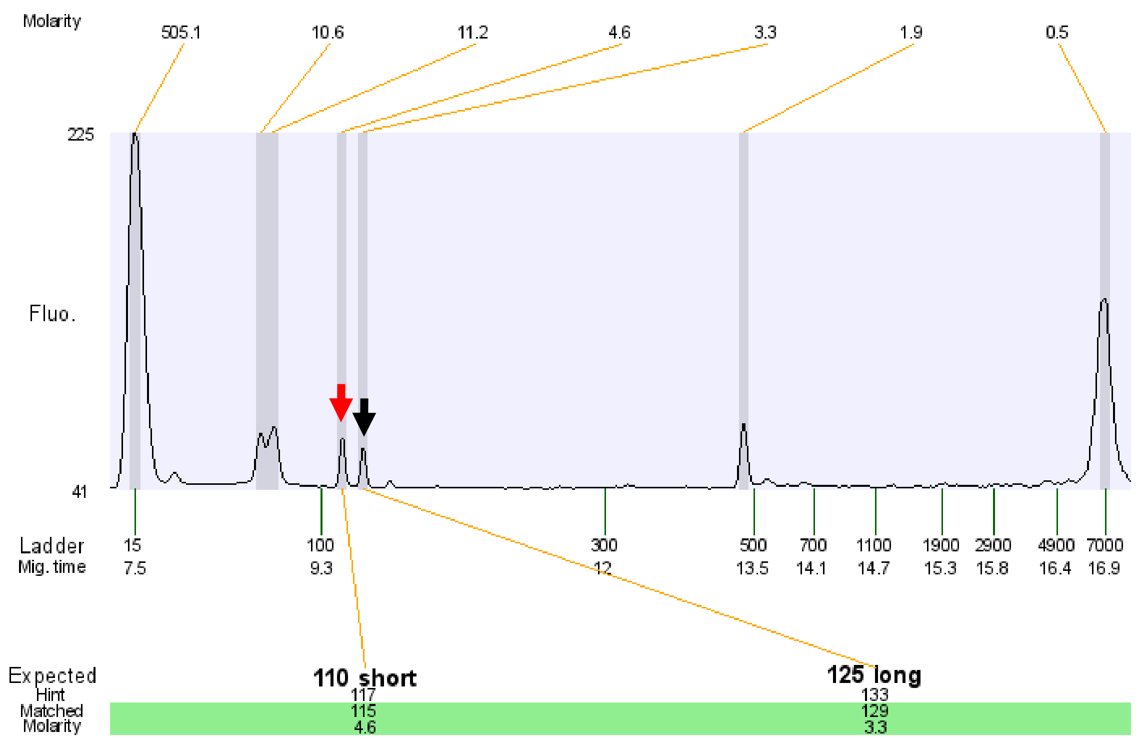


HEK293T-EBNA1

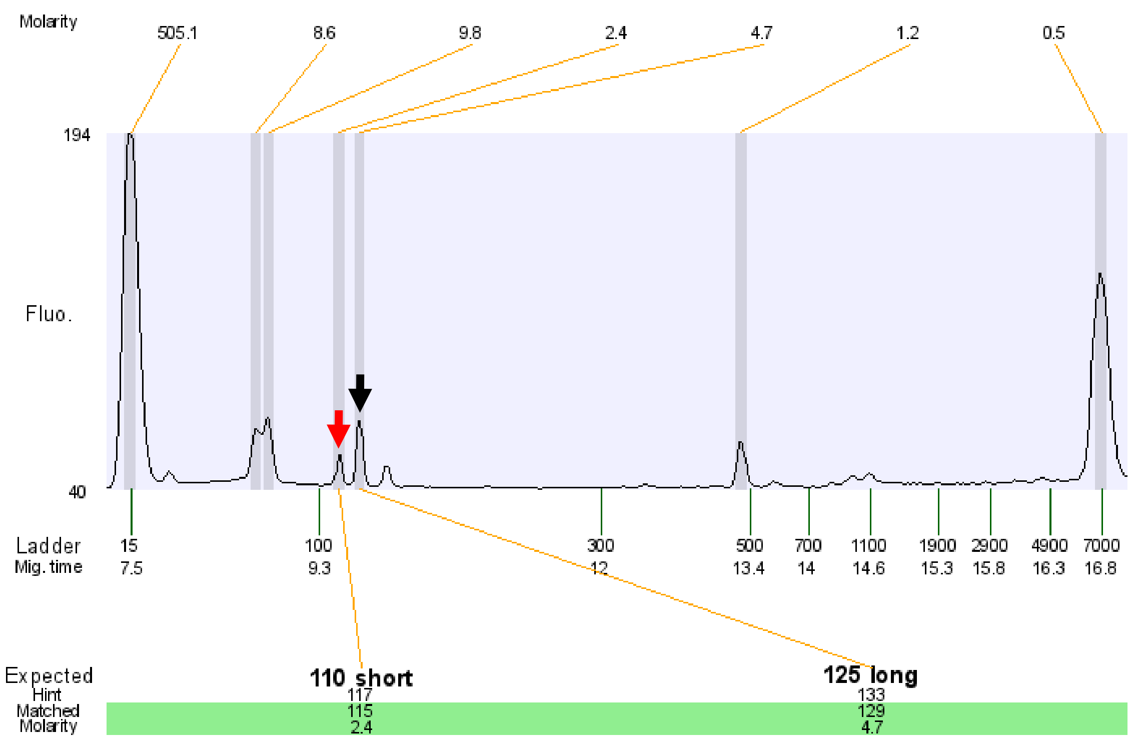


Figure S1 (cont'd)

# IL37 (ΔPSI=-38.6)

HEK293T

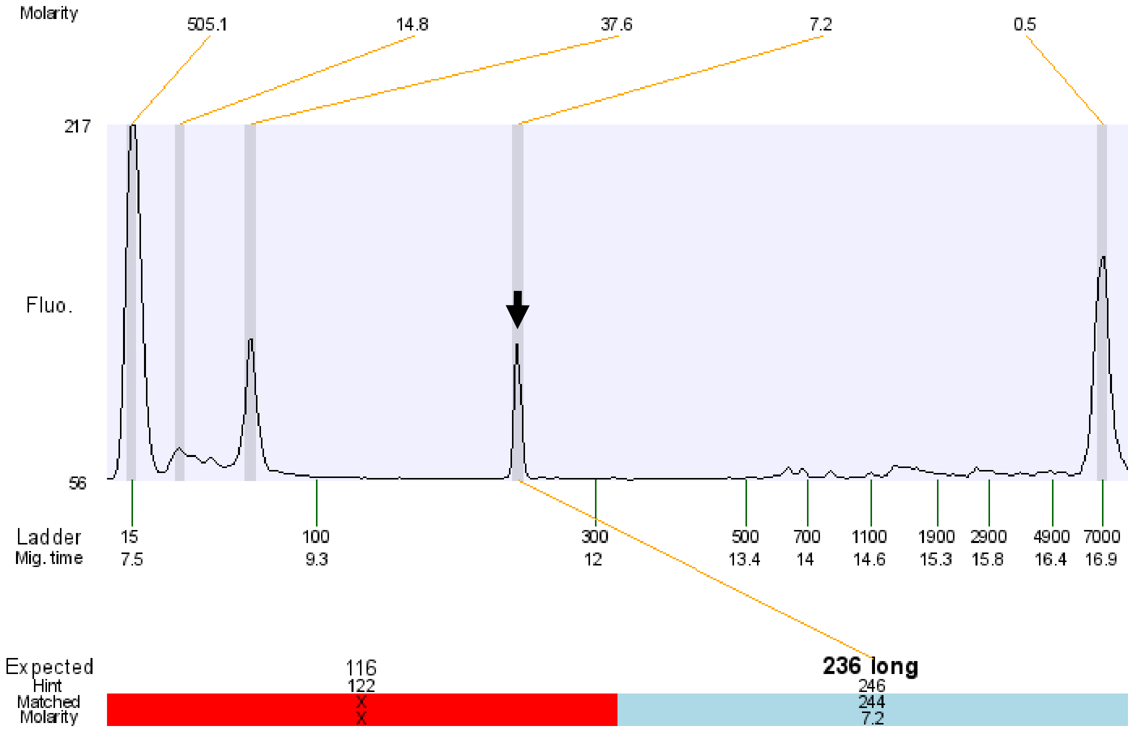


HEK293T-EBNA1

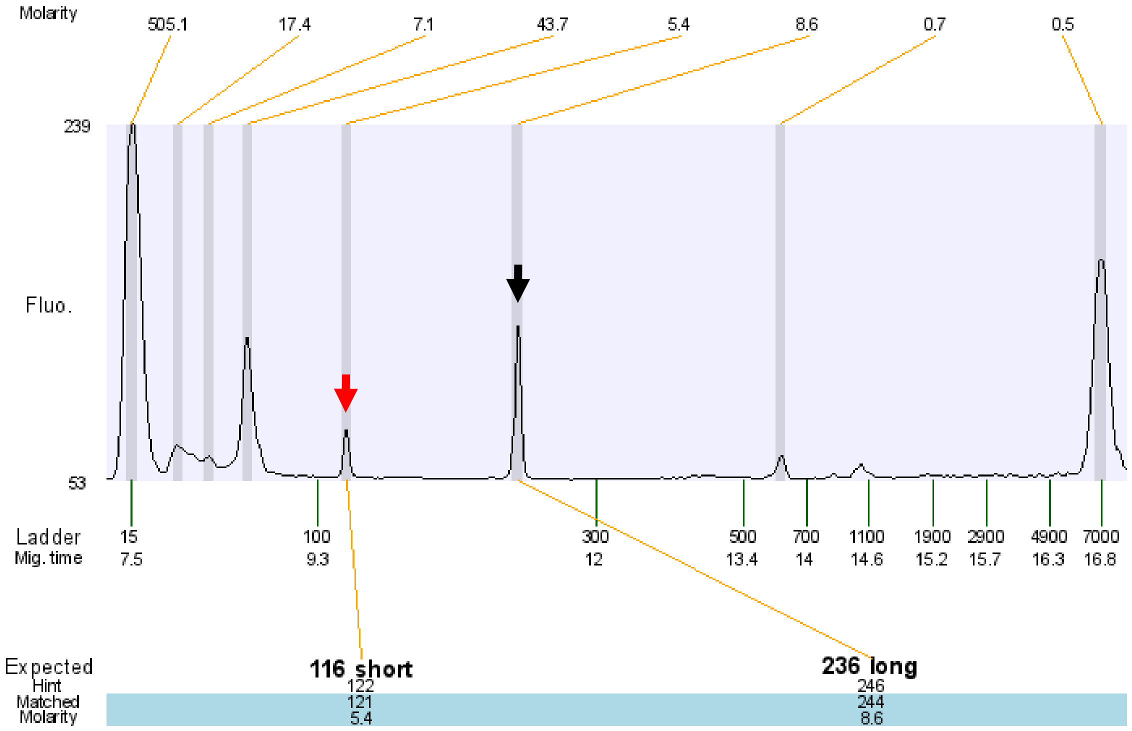


Figure S1 (cont'd)

# ITGB1 (ΔPSI=-35.5)

HEK293T

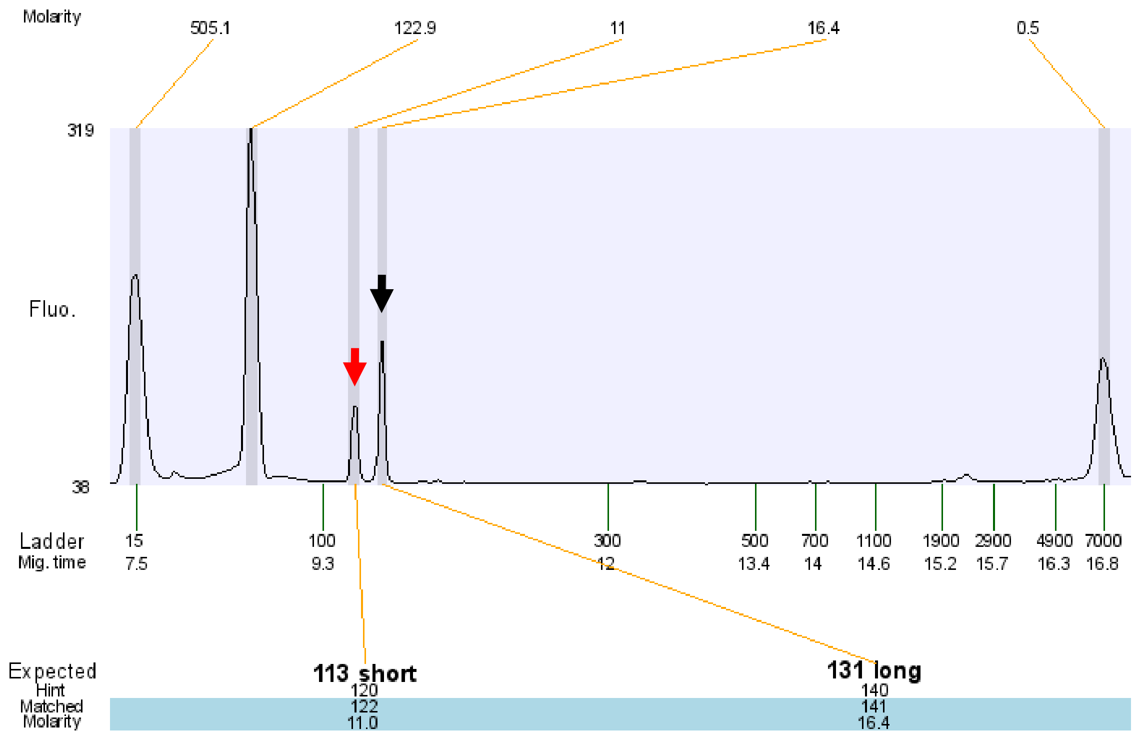


HEK293T-EBNA1

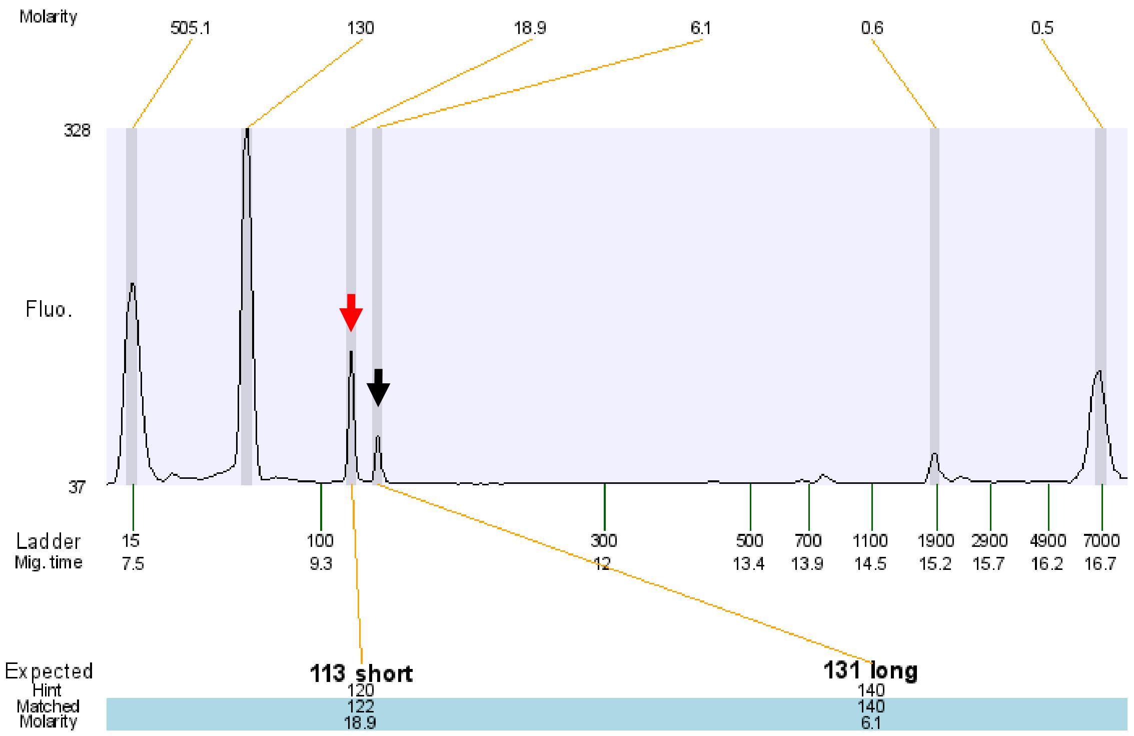


Figure S1 (cont'd)

# KIF25 (ΔPSI=42)

HEK293T

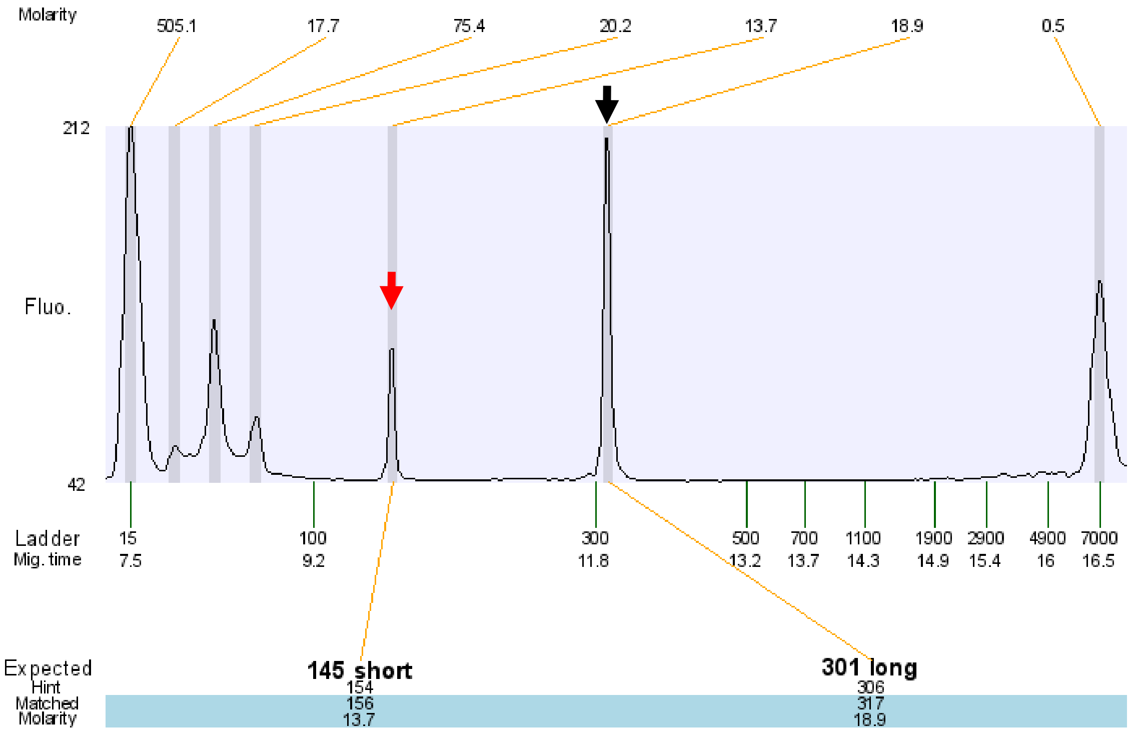


HEK293T-EBNA1

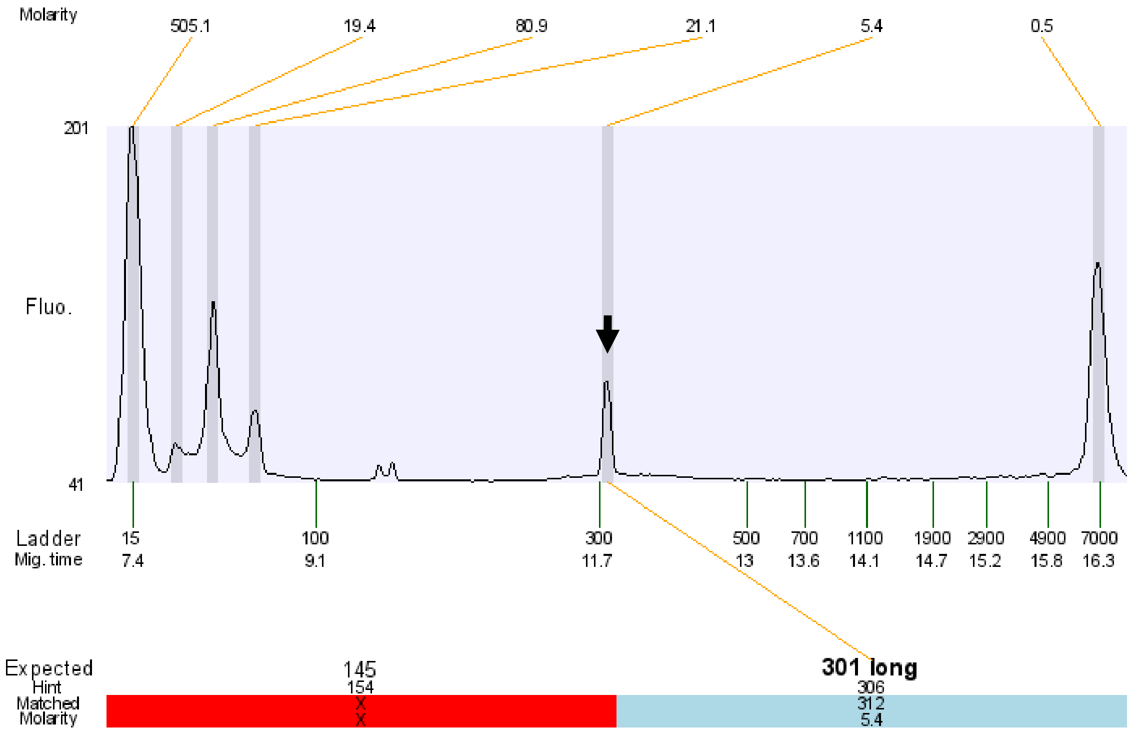


Figure S1 (cont’d)

# PAOX (ΔPSI=-73.8)

HEK293T

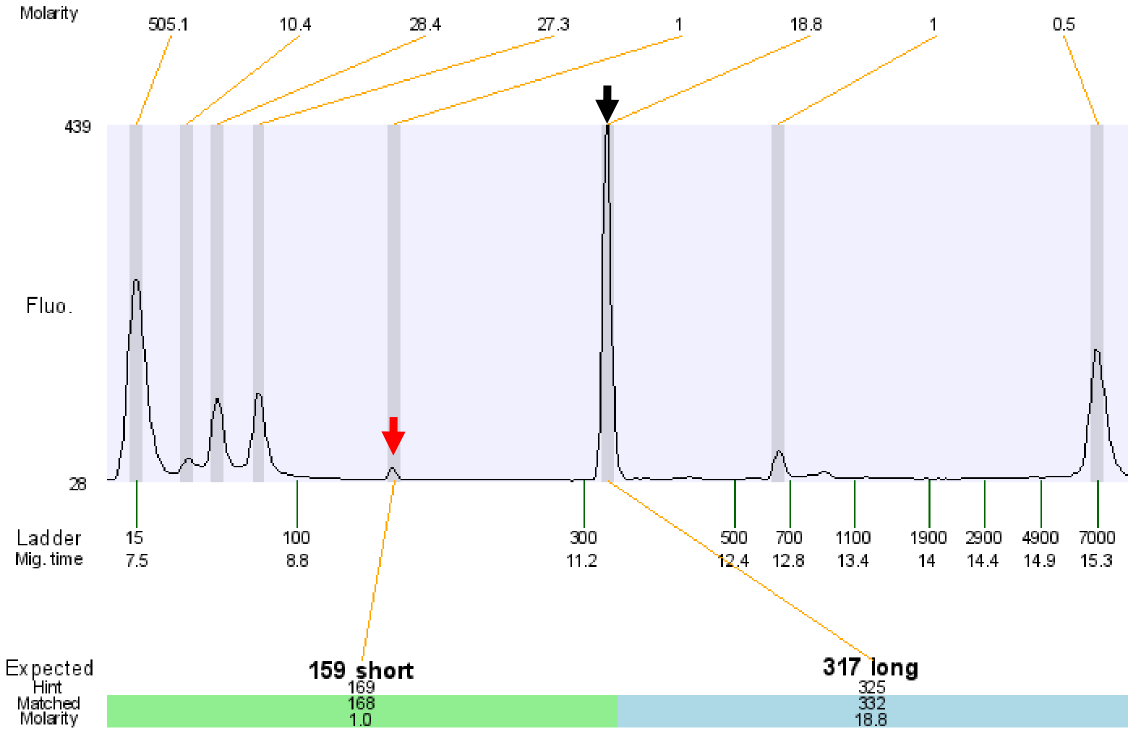


HEK293T-EBNA1

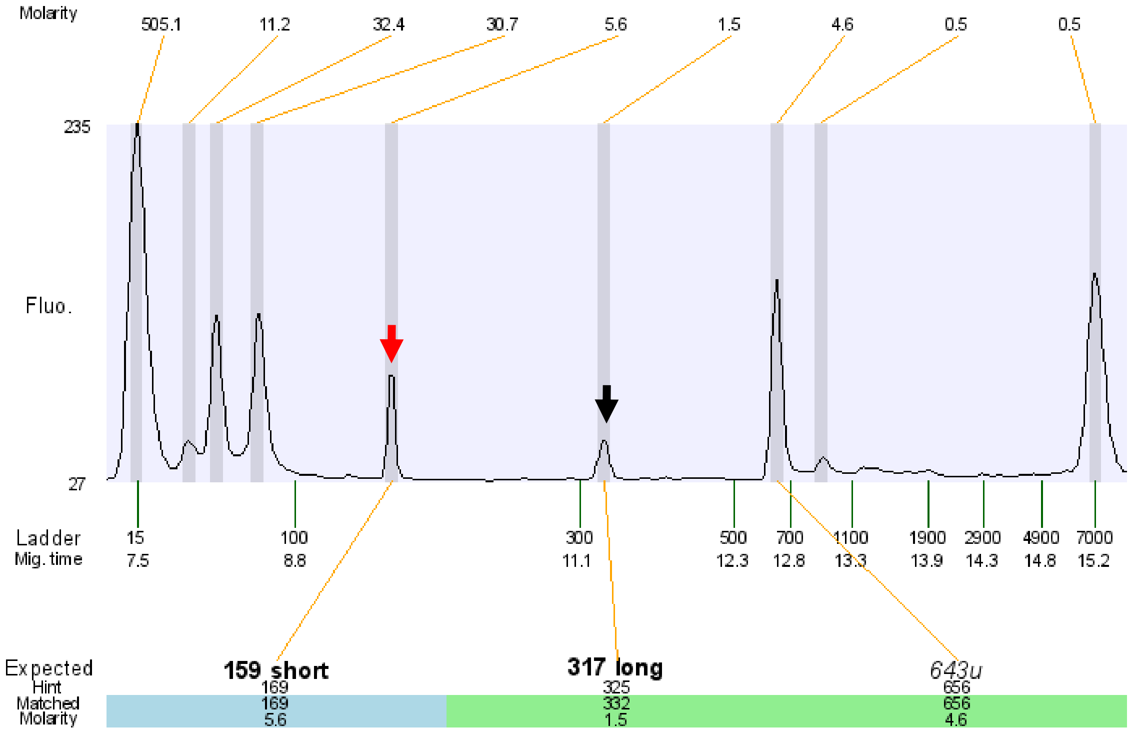


Figure S1 (cont’d)

Figure S2. Effect of transient MSCV-N transfection on cellular AS.
HEK-293T cells were transfected using Lipofectamine2000 with either no DNA or empty MSCV-N vector. RNA was harvested at 24h post-transfection and assessed for splicing using AS-specific primers in AS-PCR. Upon capillary electrophoresis of the amplicons, the PSI metric was calculated. The experiment was done in duplicate. A PSI of 0 is depicted in red, and a PSI of 1 in green, using a continuous color scale. Black depicts samples where no amplification were detectable.

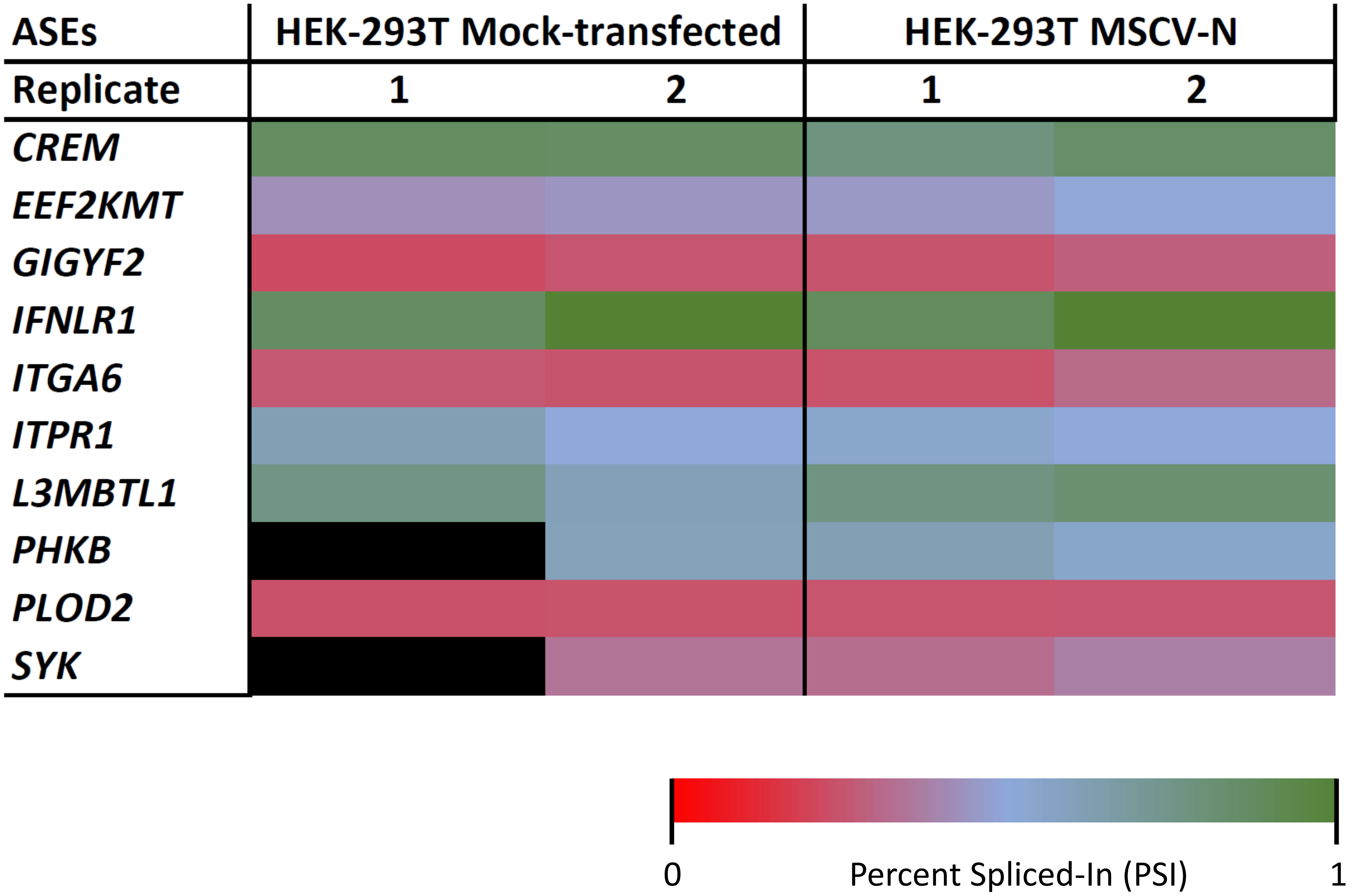
ASEs
HEK-293T Mock-transfected
HEK-293T MSCV-N
Replicate
1
2
1
2
CREM
EEF2KMT
GIGYF2
IFNLR1
ITGA6
ITPR1
L3MBTL1
PHKB
PLOD2
SYK
0
Percent Spliced-In (PSI)
1

Figure S2

Figure S3. Flowchart summarizing the RIP-Seq protocol and controls used.
The main steps of the RIP-Seq protocol are outlined, together with the controls used to validate efficient recovery of EBNA1-bound RNA with the number of their respective supplementary figures.

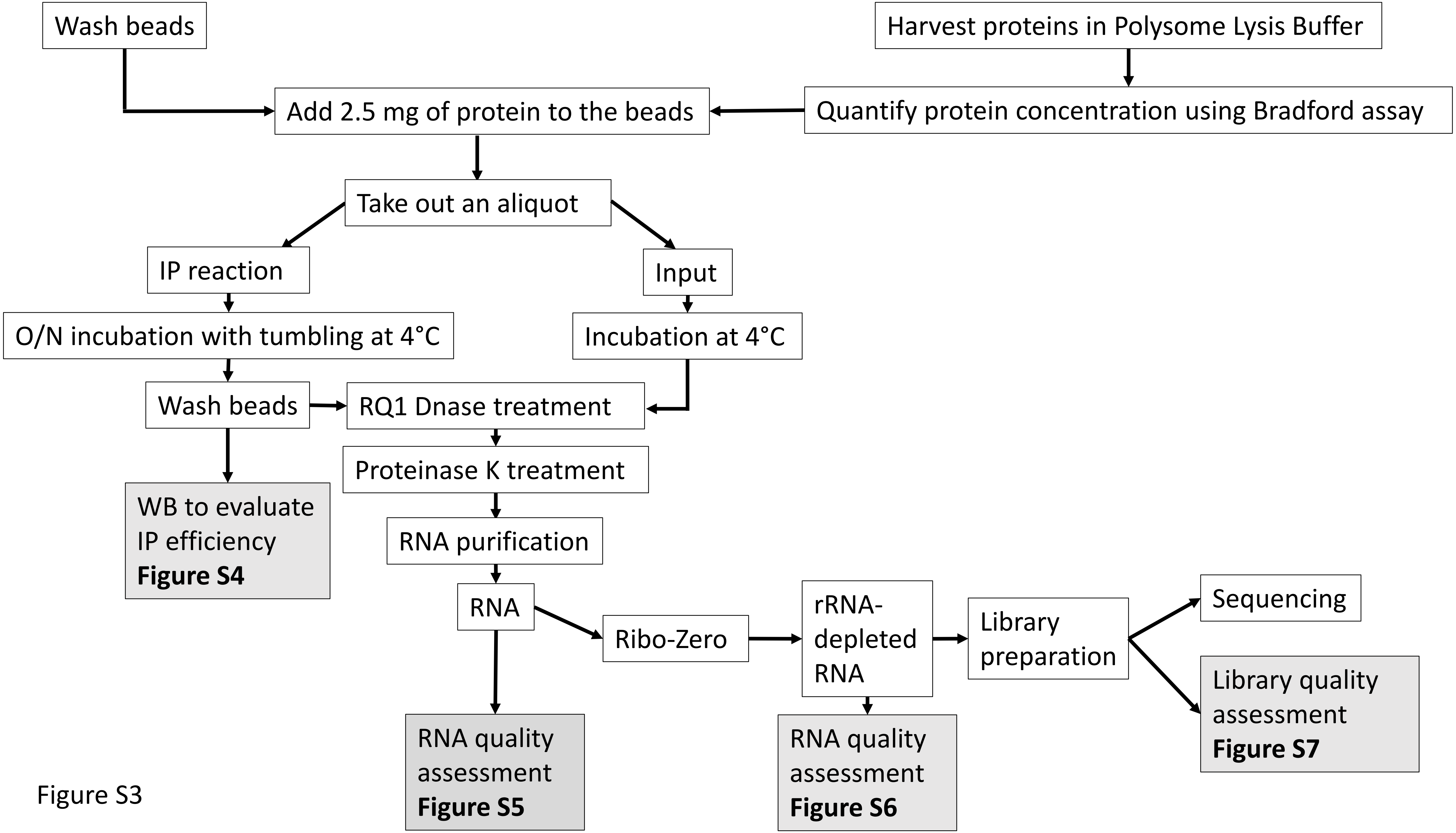
Wash beads
Harvest proteins in Polysome Lysis Buffer
Add 2.5 mg of protein to the beads
Quantify protein concentration using Bradford assay
Take out an aliquot
IP reaction
Input
O/N incubation with tumbling at 4°C
Incubation at 4°C
Wash beads
RQ1 Dnase treatment
Proteinase K treatment
WB to evaluate IP efficiency
Figure S4
RNA purification
RNA
Ribo-Zero
rRNA-depleted RNA
Library preparation
Sequencing
Library quality assessment
Figure S7
RNA quality assessment
Figure S5
RNA quality assessment
Figure S6

Figure S3

## Figure S4. Western-Blotting of immunoprecipitation of EBNA1.

The supernatant and beads following immunoprecipitation using an anti-HA antibody were assessed for EBNA1 levels using an anti-FLAG antibody, showing strong enrichment of EBNA1 only in the IP from EBNA1-expressing cells.

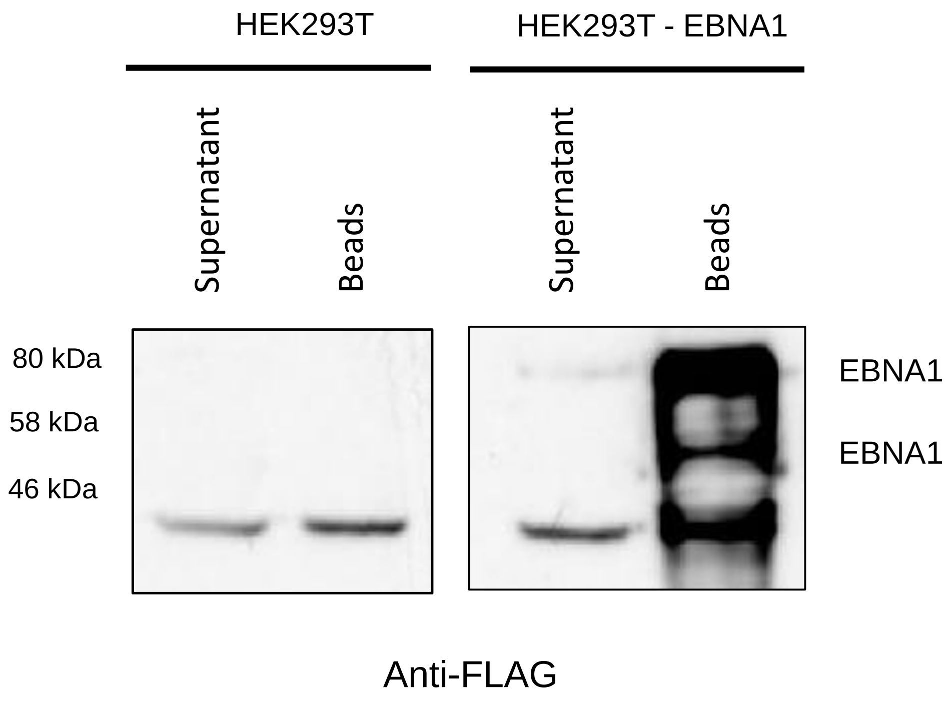
HEK293T
HEK293T - EBNA1
Supernatant
Beads
Supernatant
Beads
80 kDa
58 kDa
46 kDa
EBNA1
EBNA1
Anti-FLAG

Figure S4

Figure S5. Quality assessment of input RNA and immunoprecipitated RNA for the RIP-Seq experiment.
RNA integrity number (RIN) and global quality of RNA were determined using an Agilent Nano Chip.

Input | RIP

## HEK293T

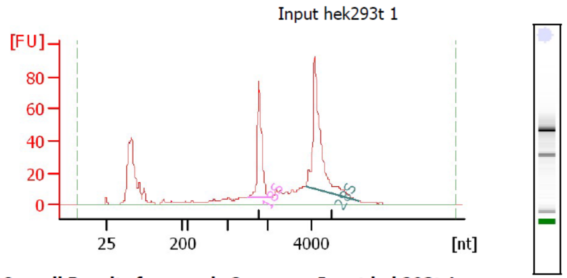


**Overall Results for sample 3 : Input hek293t 1**

| | |
|---|---|
| RNA Area: | 785,9 |
| RNA Concentration: | 1 265 ng/µl |
| rRNA Ratio [28s / 18s]: | 1,8 |
| RNA Integrity Number (RIN): | 8.9 (B.02.08) |
| Result Flagging Color: | |
| Result Flagging Label: | RIN: 8.90 |

**Fragment table for sample 3 : Input hek293t 1**

| Name | Start Size [nt] | End Size [nt] | Area | % of total Area |
|---|---|---|---|---|
| 18S | 1 477 | 2 150 | 107,2 | 13,6 |
| 28S | 3 685 | 6 133 | 196,6 | 25,0 |

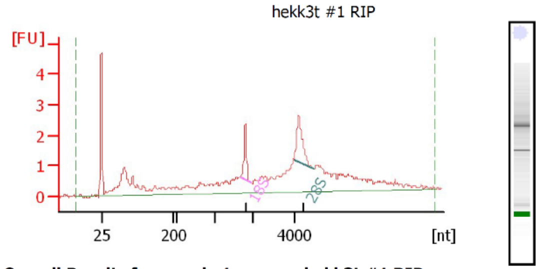


**Overall Results for sample 1 : hekk3t #1 RIP**

| | |
|---|---|
| RNA Area: | 45,1 |
| RNA Concentration: | 73 ng/µl |
| rRNA Ratio [28s / 18s]: | 1,5 |
| RNA Integrity Number (RIN): | 5.7 (B.02.08) |
| Result Flagging Color: | |
| Result Flagging Label: | RIN: 5.70 |

**Fragment table for sample 1 : hekk3t #1 RIP**

| Name | Start Size [nt] | End Size [nt] | Area | % of total Area |
|---|---|---|---|---|
| 18S | 1 658 | 1 945 | 1,8 | 4,0 |
| 28S | 3 956 | 4 863 | 2,8 | 6,2 |

## HEK293T-EBNA1

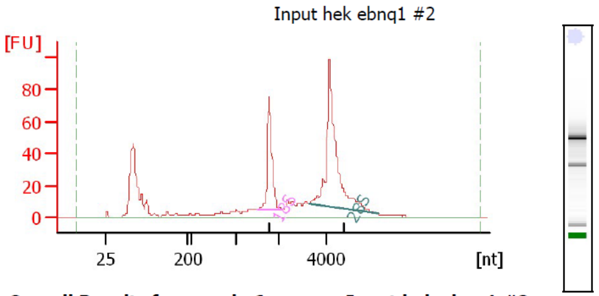


**Overall Results for sample 6 : Input hek ebnq1 #2**

| | |
|---|---|
| RNA Area: | 838,4 |
| RNA Concentration: | 1 349 ng/µl |
| rRNA Ratio [28s / 18s]: | 2,5 |
| RNA Integrity Number (RIN): | 9.2 (B.02.08) |
| Result Flagging Color: | |
| Result Flagging Label: | RIN: 9.20 |

**Fragment table for sample 6 : Input hek ebnq1 #2**

| Name | Start Size [nt] | End Size [nt] | Area | % of total Area |
|---|---|---|---|---|
| 18S | 1 532 | 2 166 | 101,7 | 12,1 |
| 28S | 3 272 | 6 192 | 254,8 | 30,4 |

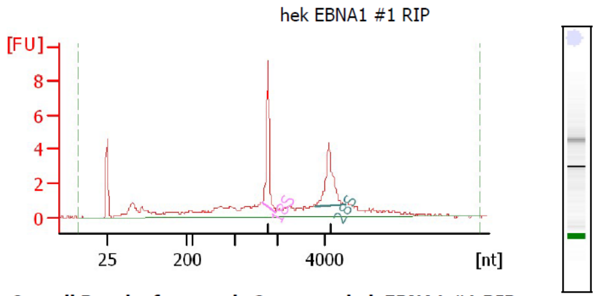


**Overall Results for sample 2 : hek EBNA1 #1 RIP**

| | |
|---|---|
| RNA Area: | 58,6 |
| RNA Concentration: | 94 ng/µl |
| rRNA Ratio [28s / 18s]: | 0,9 |
| RNA Integrity Number (RIN): | 7.4 (B.02.08) |
| Result Flagging Color: | |
| Result Flagging Label: | RIN: 7.40 |

**Fragment table for sample 2 : hek EBNA1 #1 RIP**

| Name | Start Size [nt] | End Size [nt] | Area | % of total Area |
|---|---|---|---|---|
| 18S | 1 660 | 1 993 | 8,2 | 14,0 |
| 28S | 3 606 | 4 910 | 7,6 | 13,0 |

Figure S5

Figure S6. Quality assessment of control and EBNA1 RIP following ribo-depletion.
Global quality of RNA following ribo-depletion was determined using an Agilent Nano Chip and underlined the complete removal of ribosomal RNA.

## Control IP
## HEK293T

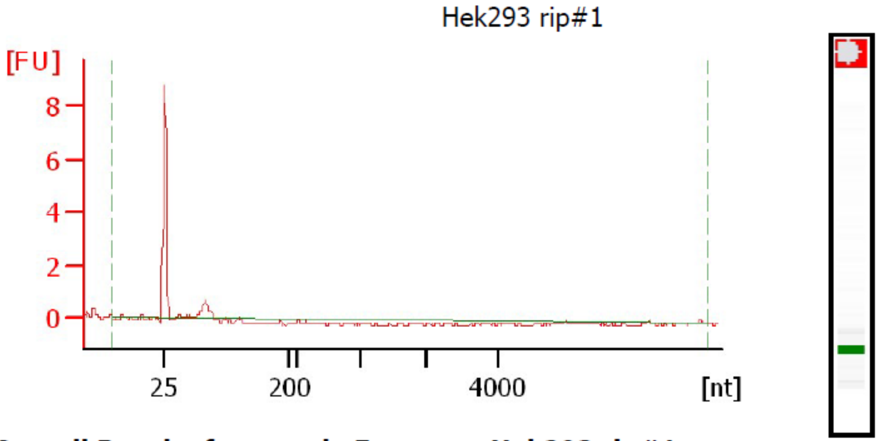


**Overall Results for sample 5 :** **Hek293 rip#1**

| | |
|---|---|
| RNA Area: | 1,9 |
| RNA Concentration: | 2 ng/µl |
| rRNA Ratio [28s / 18s]: | 0,0 |
| RNA Integrity Number (RIN): | N/A (B.02.08) |
| Result Flagging Color: | |
| Result Flagging Label: | RIN N/A |

## EBNA1 IP
## HEK293T-EBNA1

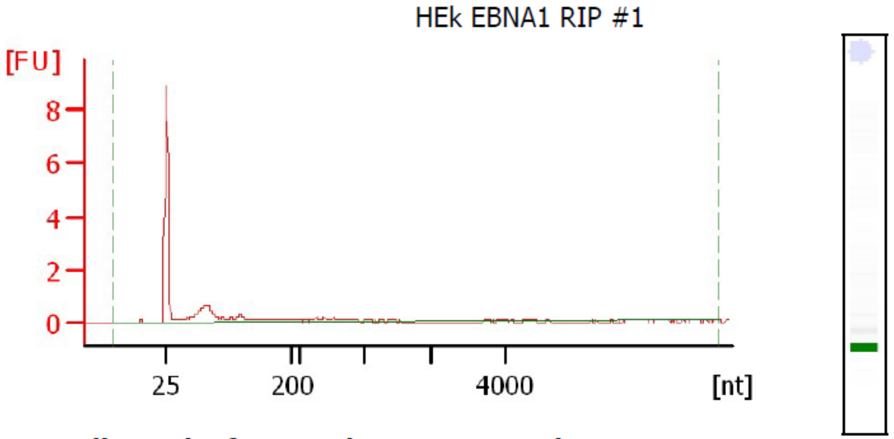


**Overall Results for sample 6 :** **HEk EBNA1 RIP #1**

| | |
|---|---|
| RNA Area: | 9,6 |
| RNA Concentration: | 11 ng/µl |
| rRNA Ratio [28s / 18s]: | 0,0 |
| RNA Integrity Number (RIN): | 1 (B.02.08) |
| Result Flagging Color: | |
| Result Flagging Label: | RIN:1 |

Figure S6

Figure S7. Quality assessment of library for the RIP-Seq.
Library quality was assessed using an Agilent DNA HS Chip.

## Control IP library HEK293T

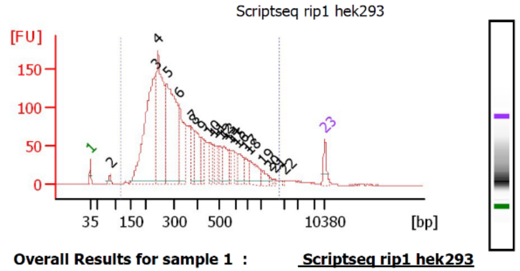


**Overall Results for sample 1 :** **Scriptseq rip1 hek293**

Number of peaks found: 21
Noise: 0,2
Corr. Area 1: 3 862,0

**Peak table for sample 1 :** **Scriptseq rip1 hek293**

| Peak | Size [bp] | Conc. [pg/µl] | Molarity [pmol/l] | Observations |
|---|---|---|---|---|
| 1 | 35 | 125,00 | 5 411,3 | Lower Marker |
| 2 | 85 | 58,71 | 1 045,8 | |
| 3 | 235 | 1 960,65 | 12 635,6 | |
| 4 | 244 | 1 539,98 | 9 568,3 | |
| 5 | 276 | 1 430,31 | 7 841,3 | |
| 6 | 324 | 604,41 | 2 827,8 | |
| 7 | 375 | 179,30 | 724,8 | |
| 8 | 387 | 352,21 | 1 378,9 | |
| 9 | 419 | 144,11 | 521,1 | |
| 10 | 456 | 106,89 | 355,0 | |
| 11 | 480 | 143,24 | 451,8 | |
| 12 | 511 | 93,89 | 278,6 | |
| 13 | 528 | 180,61 | 518,2 | |
| 14 | 561 | 84,00 | 226,9 | |
| 15 | 592 | 87,66 | 224,4 | |
| 16 | 620 | 164,34 | 401,6 | |
| 17 | 690 | 75,24 | 165,2 | |
| 18 | 759 | 180,06 | 359,5 | |
| 19 | 1 015 | 56,66 | 84,6 | |
| 20 | 1 352 | 12,24 | 13,7 | |
| 21 | 1 570 | 7,37 | 7,1 | |
| 22 | 1 997 | 6,97 | 5,3 | |
| 23 | 10 380 | 75,00 | 10,9 | Upper Marker |

## EBNA1 IP library HEK293T-EBNA1

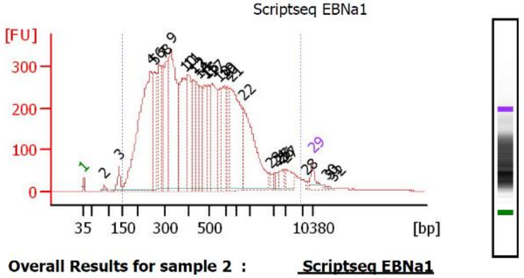


**Overall Results for sample 2 :** **Scriptseq EBNa1**

Number of peaks found: 30
Noise: 0,2
Corr. Area 1: 12 844,3

**Peak table for sample 2 :** **Scriptseq EBNa1**

| Peak | Size [bp] | Conc. [pg/µl] | Molarity [pmol/l] | Observations |
|---|---|---|---|---|
| 1 | 35 | 125,00 | 5 411,3 | Lower Marker |
| 2 | 87 | 43,40 | 755,3 | |
| 3 | 136 | 150,65 | 1 680,2 | |
| 4 | 247 | 2 523,68 | 15 455,9 | |
| 5 | 260 | 583,87 | 3 396,5 | |
| 6 | 279 | 639,40 | 3 475,6 | |
| 7 | 292 | 338,65 | 1 758,4 | |
| 8 | 309 | 591,81 | 2 898,8 | |
| 9 | 327 | 1 479,38 | 6 863,8 | |
| 10 | 386 | 750,60 | 2 948,0 | |
| 11 | 399 | 673,21 | 2 559,5 | |
| 12 | 427 | 335,51 | 1 190,9 | |
| 13 | 437 | 240,29 | 832,7 | |
| 14 | 451 | 378,62 | 1 271,8 | |
| 15 | 481 | 369,48 | 1 163,0 | |
| 16 | 495 | 334,39 | 1 023,1 | |
| 17 | 519 | 612,90 | 1 790,1 | |
| 18 | 567 | 265,26 | 708,6 | |
| 19 | 590 | 301,01 | 773,4 | |
| 20 | 609 | 242,64 | 604,0 | |
| 21 | 648 | 842,92 | 1 969,8 | |
| 22 | 841 | 840,17 | 1 513,9 | |
| 23 | 1 897 | 19,81 | 15,8 | |
| 24 | 2 116 | 22,93 | 16,4 | |
| 25 | 2 412 | 28,66 | 18,0 | |
| 26 | 2 781 | 48,76 | 26,6 | |
| 27 | 3 179 | 82,35 | 39,2 | |
| 28 | 8 175 | 9,96 | 1,8 | |
| 29 | 10 380 | 75,00 | 10,9 | Upper Marker |
| 30 | 14 790 | 0,00 | 0,0 | |
| 31 | 15 950 | 0,00 | 0,0 | |
| 32 | 16 995 | 0,00 | 0,0 | |

Figure S7

Figure S8: Read distribution on the EBNA1 coding sequence in the EBNA1 RIP-Seq and qPCR mesurement of immunoprecipitated EBNA1 mRNA.

A) Reads mapping to the EBNA1 coding sequence were retrieved using custom command line following mapping using the human reference genome and the EBNA1 coding sequence. Visualization was done using the Integrative Genomic Viewer (IGV). Red boxes represent chosen amplicons to validate EBNA1 binding site in qPCR.

B) qPCR validation of specific enriched regions from the EBNA1 coding sequence following EBNA1 immunoprecipitation. Experiments were done in triplicate and failed to show any enrichment in a particular region of the coding sequence following EBNA1 immunoprecipitation.

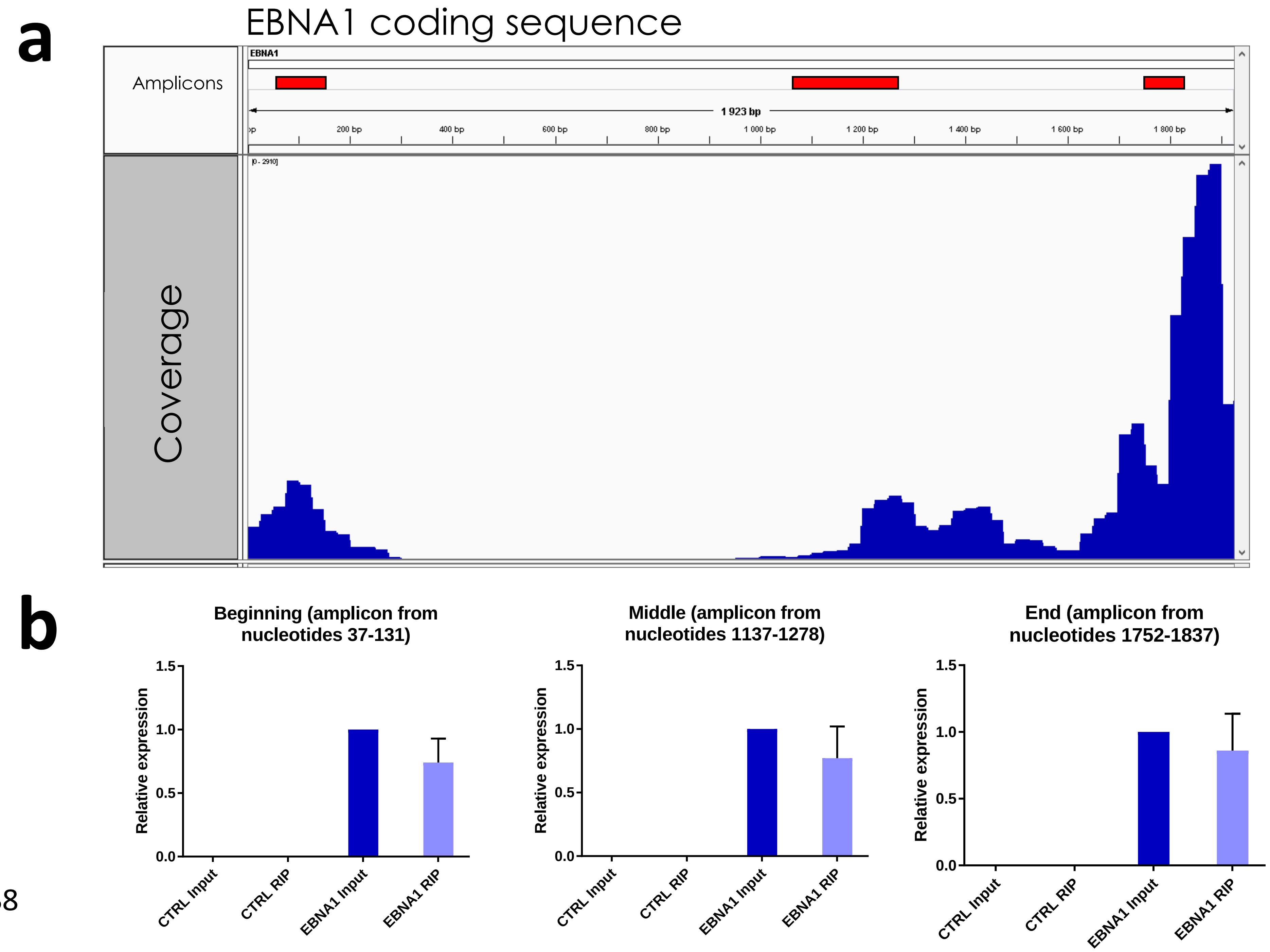
a
EBNA1 coding sequence
Amplicons
EBNA1
1 923 bp
200 bp
400 bp
600 bp
800 bp
1 000 bp
1 200 bp
1 400 bp
1 600 bp
1 800 bp
Coverage
b
Beginning (amplicon from nucleotides 37-131)
Middle (amplicon from nucleotides 1137-1278)
End (amplicon from nucleotides 1752-1837)
Relative expression
0.0
0.5
1.0
1.5
CTRL Input
CTRL RIP
EBNA1 Input
EBNA1 RIP

Figure S8
